# Reversal of MPPa‐PDT Resistance in Osteosarcoma by Targeting ROCK2‐Mediated Autophagy

**DOI:** 10.1111/cpr.70097

**Published:** 2025-07-16

**Authors:** Xuan Yi, Xueqiang Deng, Jianyong Deng, Chen Li, Hong Peng, Yunyan Du, Qing Li, Xiaohua Yan, Xin Hu, Yan Zheng, Shenliang Chen, Ting Xiong, Debin Xu, Leifeng Chen, Liang Hao

**Affiliations:** ^1^ Department of Orthopedics The Second Affiliated Hospital of Nanchang University Nanchang China; ^2^ Institute of Orthopedics of Jiangxi Province Nanchang China; ^3^ Jiangxi Provincial Key Laboratory of Spine and Spinal Cord Disease Nanchang China; ^4^ Institute of Minimally Invasive Orthopedics Nanchang University Nanchang China; ^5^ Department of Rehabilitation The Second Affiliated Hospital of Nanchang University Nanchang China; ^6^ Department of Vascular & Interventional & Lymphatic Surgery The First Hospital of Nanchang Nanchang China; ^7^ Department of Anorectal Surgery Changcheng Hospital Affiliated to Nanchang University Nanchang China; ^8^ Department of Anorectal Surgery No 908th Hospital of Chinese Nanchang China; ^9^ Department of Pharmacology School of Pharmaceutical Science, Nanchang University Nanchang China; ^10^ Department of Pathology The Second Affiliated Hospital of Nanchang University Nanchang China; ^11^ The MOE Basic Research and Innovation Center for the Targeted Therapeutics of Solid Tumors, School of Basic Medical Sciences Jiangxi Medical College, Nanchang University Nanchang China; ^12^ Department of Orthopedics Pingxiang People's Hospital PingXiang China; ^13^ Department of Medical Genetics The Second Affiliated Hospital of Nanchang University Nanchang China; ^14^ Department of General Surgery The Second Affiliated Hospital of Nanchang University Nanchang China; ^15^ Department of Oncology The Second Affiliated Hospital of Nanchang University Nanchang China; ^16^ Medical Center for Cardiovascular Diseases, Neurological Diseases and Tumors of Jiangxi Province The Second Affiliated Hospital of Nanchang University Nanchang China

**Keywords:** autophagy, hippo pathway, MPPa‐PDT, osteosarcoma, ROCK2

## Abstract

Osteosarcoma (OS) is a primary bone tumour that occurs mostly in adolescents and is associated with a high degree of malignancy, early metastasis, and poor prognosis. Pyropheophorbide‐a methyl ester‐Photodynamic therapy (MPPa‐PDT) is a new approach for the clinical treatment of osteosarcoma that develops after surgery and radiotherapy; however, the presence of MPPa‐PDT resistance in osteosarcoma greatly limits its efficacy. In this study, we found that Rho‐associated coiled‐coil containing protein kinase 2 (ROCK2) expression increased in osteosarcoma cells after MPPa‐PDT treatment. ROCK2 inhibition results in osteosarcoma sensitivity to MPPa‐PDT and is accompanied by a decrease in cellular autophagy levels. Rescue experiments further showed that ROCK2 mediates MPPa‐PDT resistance in osteosarcoma by regulating autophagy. Mechanistic studies have shown that ROCK2 mediates autophagy in osteosarcoma cells by regulating the Hippo signalling pathway. ROCK2 overexpression resulted in increased levels of the ROCK2‐Salvador homology 1 (SAV1) complex and decreased levels of the mammalian STE20‐like protein kinase 1 (MST1)‐SAV1 complex, thereby inhibiting activation of the Hippo pathway, which in turn led to osteosarcoma MPPa‐PDT resistance by regulating cellular autophagy. ROCK2 competes with MST1 for binding to the aa 28–198 region of SAV1. We also confirmed from a clinical perspective that ROCK2 is an independent prognostic factor in patients with osteosarcoma, is associated with worse patient prognosis, and correlates with the Hippo pathway. Targeted inhibition of ROCK2 by screening for J059‐0149 increases the sensitivity of osteosarcoma to MPPa‐PDT. In conclusion, our study establishes a novel mechanism to reverse MPPa‐PDT resistance in osteosarcoma by targeting ROCK2‐mediated autophagy, providing new targets and research ideas for the clinical treatment of osteosarcoma MPPa‐PDT resistance.

## Graphical abstract

ROCK2 competes with MST1 to bind to the aa 28‐198 region of SAV1 and then regulates the Hippo pathway to affect autophagy, resulting in the resistance of OS to MPPa‐PDT. Targeted inhibition of ROCK2 by screening for J059‐0149 increases the sensitivity of osteosarcoma to MPPa‐PDT.

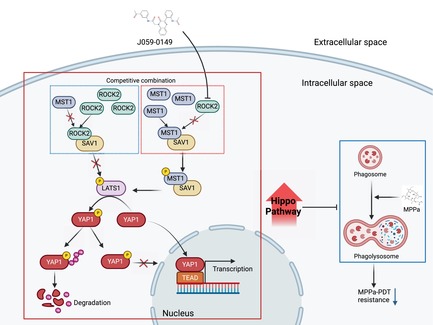

## 1 Introduction

Osteosarcoma (OS) is a primary malignant tumour of bone and the second most prevalent primary bone tumour after plasma cell myeloma. The features of OS include a high prevalence among adolescents, a high degree of malignancy, early metastasis, and markedly poor prognosis [1]. Since the introduction of effective adjuvant chemotherapy in the 1970s, particularly the development of neoadjuvant chemotherapy combined with traditional limb salvage surgery has greatly improved the quality of life and 5‐year survival rate of patients with OS [2, 3]. However, despite significant advances in the treatment of OS in recent years, approximately 30% of patients remain chemotherapy‐resistant leading to a higher risk of lung metastases, while complete tumour resection does not significantly improve their prognosis.

Photodynamic therapy (PDT) has achieved remarkable research results on clinical trials of malignant tumours, mainly skin, lung, oesophageal, and head–neck tumours [4]. Because OS tends to develop in the distal femur and proximal tibia, where the tumour is superficial, treatment with PDT can reduce skin and muscle damage [5]. Studies have confirmed that PDT combined with surgery and chemotherapy can greatly reduce local tumour recurrence, maintain good limb function, and effectively kill tumour cells that have spread in the surgical area [6, 7]. Pyropheophorbide‐a methyl ester (MPPa), a second‐generation photosensitiser derived from chlorophyll, exhibits strong photosensitivity, is easily absorbed, rapidly metabolised, and possesses stable properties [8, 9]. Previous studies have demonstrated that MPPa‐PDT induces apoptosis in OS cells [8, 10]. However, the resistance of OS to MPPa‐PDT in clinical applications considerably limits its effectiveness, and the underlying reasons for this resistance are unclear [10, 11]. Therefore, elucidating the mechanism of MPPa‐PDT resistance is crucial for developing more effective therapeutic strategies and enhancing the anti‐tumour effects of MPPa‐PDT in OS.

Autophagy, also known as type II programmed cell death, is a lysosome‐dependent degradation pathway widely found in eukaryotic cells and a programmed cellular self‐repair process [12, 13]. The autolysosome degradation pathway plays a crucial role in maintaining cellular homeostasis by eliminating excessive or damaged organelles such as peroxisomes, endoplasmic reticulum, mitochondria, and abnormal DNA and protein aggregates [14]. Studies have shown that autophagy may significantly impact the therapeutic efficacy of PDT against tumours. While PDT primarily relies on Reactive Oxygen Species (ROS) to trigger tumour cell death and apoptosis, autophagy can provide cytoprotection to tumour cells by scavenging ROS and inhibiting apoptotic pathways [9, 15, 16, 17]. Therefore, autophagy may delay the onset of apoptosis by eliminating ROS and damaged organelles during MPPa‐PDT, thereby exerting a protective mechanism that renders tumour cells resistant to MPPa‐PDT. Repressing autophagy may offer novel strategies for reversing MPPa‐PDT resistance in OS.

Rho‐associated coiled‐coil containing protein kinase 2 (ROCK2) is a pivotal signalling molecule in the Rho/ROCK pathway, located on chromosome 2P24, encoding 1338 amino acids, which regulates various essential cellular functions, such as contraction, adhesion, migration, proliferation, and apoptosis [18, 19]. Studies have confirmed the crucial role of ROCK2 in regulating the actin‐myosin cytoskeleton. ROCK2 promotes actin bunding by activating and crosslinking myosin, thereby participating in the regulation of physiological functions such as cell morphology, polarity, motility, and cytoskeletal remodelling [20, 21]. Moreover, ROCK2 has been demonstrated to exert a pivotal role in tumourigenesis [22]. ROCK2 is highly expressed in various types of tumours, including liver cancer [23], breast cancer [24], and lung cancer [25]. Repression of ROCK2 expression inhibits cancer initiation and progression [18]. Studies have demonstrated that ROCK2 mediates the malignant transformation of gastric cancer and cisplatin resistance through the regulation of cellular autophagy [26]. However, there is a paucity of studies investigating the correlation between ROCK2 and autophagy in OS, and the underlying mechanisms remain unclear and warrant further investigation.

The Hippo signalling pathway is a highly conserved growth control pathway that regulates cell proliferation, apoptosis, organ development and growth, and cell contact inhibition [27]. The classical Hippo signalling pathway transduction in mammals can be summarised as follows: Salvador homology 1 (SAV1) and MOB kinase activator 1 (MOB1) act as scaffold proteins that facilitate the phosphorylation of mammalian STE20‐like protein kinase 1/2 (MST1/2) and large tumour suppressor serine/threonine protein kinases 1/2 (LATS1/2), respectively. Upstream signalling molecules activate MST1/2 kinase, MST1/2 co‐phosphorylates and activates LATS1/2 with scaffold protein SAV1, and activated LATS1/2 kinase co‐phosphorylates transcriptional co‐activator Yes‐associated protein (YAP)/transcriptional co‐activator with PDZ‐binding motif (TAZ) with MOB1. Phosphorylated YAP/TAZ is either retained in the cytoplasm or degraded via ubiquitination. Conversely, unphosphorylated YAP/TAZ enters the nucleus and interacts with downstream signalling molecules to exert transcription factor effects, thereby promoting the transcription of target genes and enhancing cell proliferation, metastasis, and invasion [28, 29, 30, 31]. Numerous recent studies have confirmed the close relationship between the Hippo pathway and autophagy, with Hippo pathway‐mediated autophagy playing a crucial role in cancer therapy and drug resistance [32, 33, 34]. However, the effect of the Hippo pathway on autophagy in OS remains unclear, and whether it regulates PDT resistance by mediating autophagy in OS requires further study.

In the present study, we found that ROCK2 is closely related to MPPa‐PDT resistance of OS. MPPa‐PDT induces upregulation of ROCK2 expression in OS cells, with ROCK2‐mediated autophagy involved in the resistance of OS to MPPa‐PDT. Specifically, ROCK2 regulates the Hippo signalling pathway by competing with MST1 for binding to SAV1, thereby modulating autophagy and ultimately inducing the resistance of OS cells to MPPa‐PDT. In addition, we also identified ROCK2 as an independent prognostic factor in patients with OS from a clinical perspective, with high expression correlating with poor prognosis of the patients. ROCK2 is correlated with Hippo pathway molecules. Further, we screened for the small molecule compound J059‐0149 by targeting ROCK2, demonstrating that its inhibition of ROCK2 increased the sensitivity of OS to MPPa‐PDT. This study offers novel insights and targets for reversing MPPa‐PDT resistance in parents of OS.

## 2 Materials and Methods

### 2.1 Cell Culture and Reagents

HEK 293T, OS cell lines MG‐63, Saos‐2, U2‐OS, and 143B, as well as normal osteogenic‐derived cells hfoB1‐19 were obtained from the Shanghai Cell Bank of the Chinese Academy of Medical Sciences (Shanghai, China). The cells were cultured in DMEM, MEM, and DMEM/F12 media (Gibco, USA), supplemented with 10% FBS (Gibco, USA) and 1% penicillin–streptomycin solution (BI, Israel). Cells were incubated in a cell culture incubator at 37°C, 5% CO_2_, and 95% humidity. Antibodies against ROCK2, YAP1 (YAP), p‐YAP1 (Ser127), GAPDH, β‐actin, LATS1, MST1, Bax, and Bcl‐2 were obtained from Abcam. Antibodies against P62, LC3B, Beclin‐1, and p‐MST1/2 (T183/180) were procured from Cell Signalling Technology. Antibodies against GST and Flag were obtained from Proteintech. SAV1 antibody, GSH beads, and protein A/G PLUS‐Agarose were procured from Santa Cruz, while the antibody targeting p‐LATS1/2 (Ser909/872) was obtained from Affinity Biosciences. Pyropheophorbide‐a methyl ester (MPPa), Y27632, Rapamycin, 3‐Methyladenine (3‐MA), and cytochalasin D were purchased from MedChemExpress LCC.

### 2.2 Immunofluorescence

The Immunofluorescence assay was performed as described previously [35].

### 2.3 Cell Viability Assay

The cell viability assay was performed as described previously [36].

### 2.4 Co‐Immunoprecipitation (Co‐IP) and GST Pulldown Assay

The Co‐IP and GST pulldown assays were performed as described previously [37].

### 2.5 Statistical Analysis

All experiments were conducted at least three times and presented as mean ± standard deviation (SD). Statistical analysis of the experimental data was performed using SPSS 26 (Armonk, NY, USA) and Prism 7 software (San Diego, CA). *T*‐test was used for pairwise comparisons of quantitative data, while one‐way analysis of variance (ANOVA) was used to compare the means among multiple groups. *p* < 0.05 was considered statistically significant.

Other materials and methods, including in vitro assays and in vivo studies, are provided in the Supporting Information.

## 3 Results

### 3.1 Concentration Screening of MPPa‐PDT Treated OS Cells and ROCK2 is Closely Related to MPPa‐PDT Resistance of OS

**FIGURE 1:**
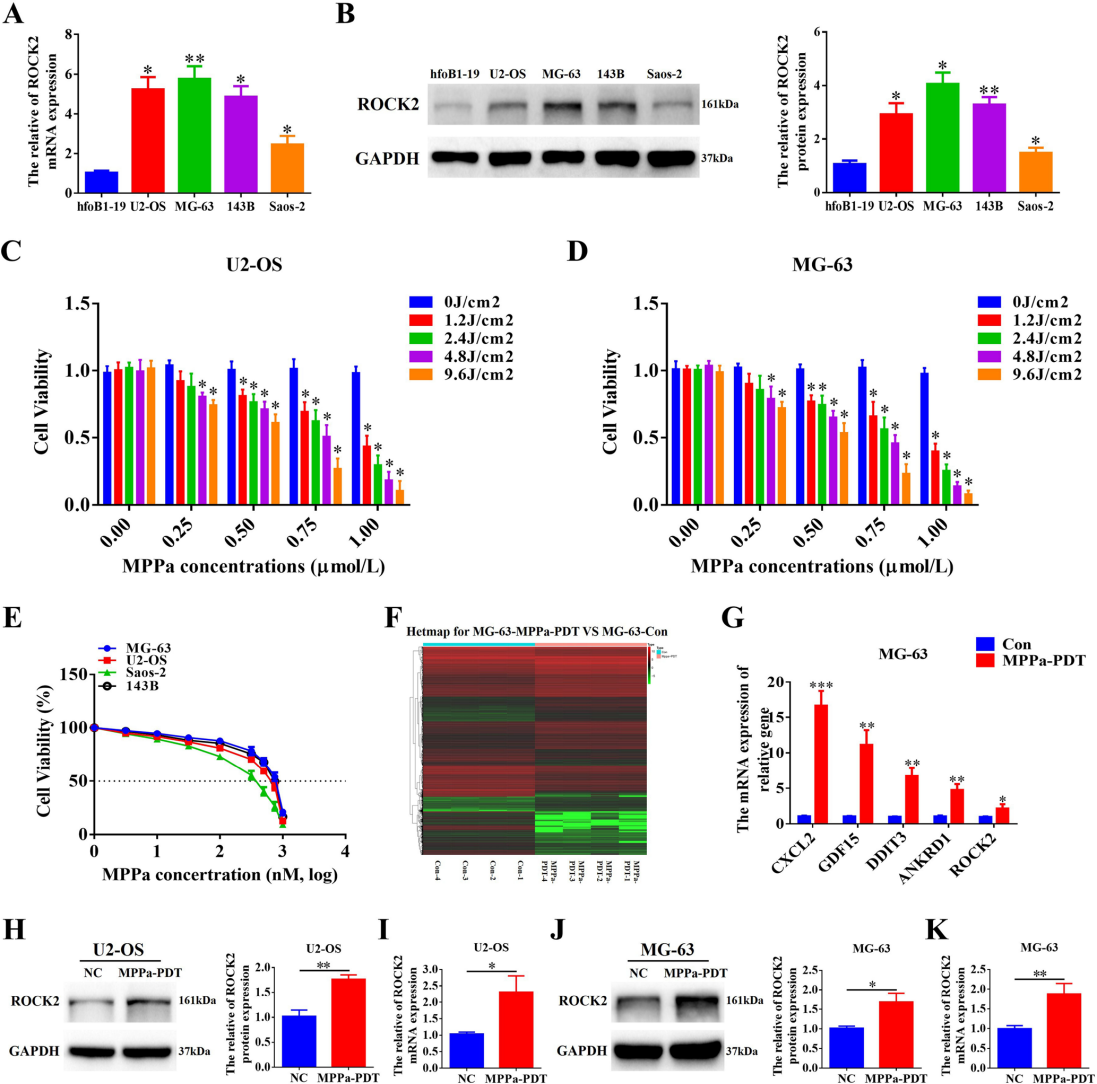
Concentration screening of MPPa‐PDT treated OS cells and ROCK2 is closely related to MPPa‐PDT resistance of OS. (A, B) The mRNA and protein expression levels of ROCK2 in OS cells (MG‐63, U2‐OS, 143B, and Saos‐2) and normal osteoblasts hfoB1‐19 were detected by qRT‐PCR and Western blot. (C, D) Different concentrations of MPPa (0, 0.25, 0.5, 0.75, and 1 μmol/L) were used to treat U2‐OS and MG‐63 cells for 24 h, and then the cells were treated with LED light of 0, 1.2, 2.4, 4.8, and 9.6 J/cm^2^ (the laser power density was 40 mW/cm^2^, the exposure times were 0, 30, 60, 120, and 240 s) energy density, respectively. Cell viability was detected using CCK‐8 after 12 h of incubation. (E) The viability of OS cells exposed to different concentrations of MPPa was analysed at a light energy density of 4.8 J/cm^2^. Inhibition curves were fitted by nonlinear regression, and IC50s were calculated using GraphPad Prism 7 software. (F) Heatmap shows differentially expressed genes. (G) qRT‐PCR detection of ROCK2 and the associated differentially highly expressed genes obtained by RNA sequencing. (H–K) Western blot and qRT‐PCR were used to detect the protein and mRNA expression levels of ROCK2 in U2‐OS and MG‐63 cells after MPPa‐PDT treatment. *p < 0.05, **p < 0.01, ***p < 0.001.

OS resistance to MPPa‐PDT exists in clinical treatments; therefore, it is important to elucidate its specific mechanism. We first analysed the expression levels of ROCK2 in OS and normal hfoB1‐19 cells. The data showed that the mRNA and protein levels of ROCK2 were significantly increased in the OS cell lines (Figure 1A,B). Based on these results, we selected U2‐OS and MG‐63 cells for subsequent experiments and further screened out the appropriate MPPa concentration and energy density of the LED light (630 nm) required for MPPa‐PDT treatment. OS cells were treated by combining different concentrations of MPPa (0, 0.25, 0.5, 0.75, and 1 μmol/L) and LED illumination with different energy densities (0, 1.2, 2.4, 4.8, and 9.6 J/cm^2^). The effects on the viability of OS cells were measured using the CCK‐8 assay (Figure 1C,D). The results revealed that neither the group with MPPa alone nor LED light alone showed significant inhibition on the viability of OS cells compared with the control group (0 μmol/L, 0 J/cm^2^). In addition to the 0.25 μmol/L MPPa combined with 1.2 J/cm^2^ or 2.4 J/cm^2^ light energy density groups, the viability of OS cells in all the other MPPa‐PDT groups was significantly inhibited. Similarly, the results showed that the viability of OS cells was inhibited in a concentration‐dependent or energy‐density‐dependent manner. According to the inhibition of OS cell viability, the 0.75 μmol/L MPPa concentration combined with 4.8 J/cm^2^ light energy density was selected for subsequent experiments. Subsequently, the viability of OS cells exposed to different MPPa concentrations was analysed at a light energy density of 4.8 J/cm^2^, and the corresponding IC50 values were obtained (Figure 1E). We found that OS cell lines with higher ROCK2 expression were more resistant to MPPa‐PDT. Meanwhile, we conducted RNA sequencing analysis on MG‐63 cells from both the MPPa‐treated and control groups. The heatmap shows the differentially expressed genes in the MPPa‐PDT group compared to those in the control group (Figure 1F), with an observed increase in the ROCK2 expression level. Moreover, qRT‐PCR detection of ROCK2 and the associated differentially highly expressed genes obtained through mRNA sequencing further confirmed that its expression was indeed elevated (Figure 1G). Following the treatment of OS cells with MPPa‐PDT, the expression level of ROCK2 in the treatment group was higher than that in the normal control group, as determined by Western blot and qRT‐PCR (Figure 1H–K). These results indicate that ROCK2 is closely related to MPPa‐PDT resistance in OS.

### 3.2 Inhibition of ROCK2 leads to sensitivity of OS to MPPa‐PDT in vivo and in vitro

**FIGURE 2:**
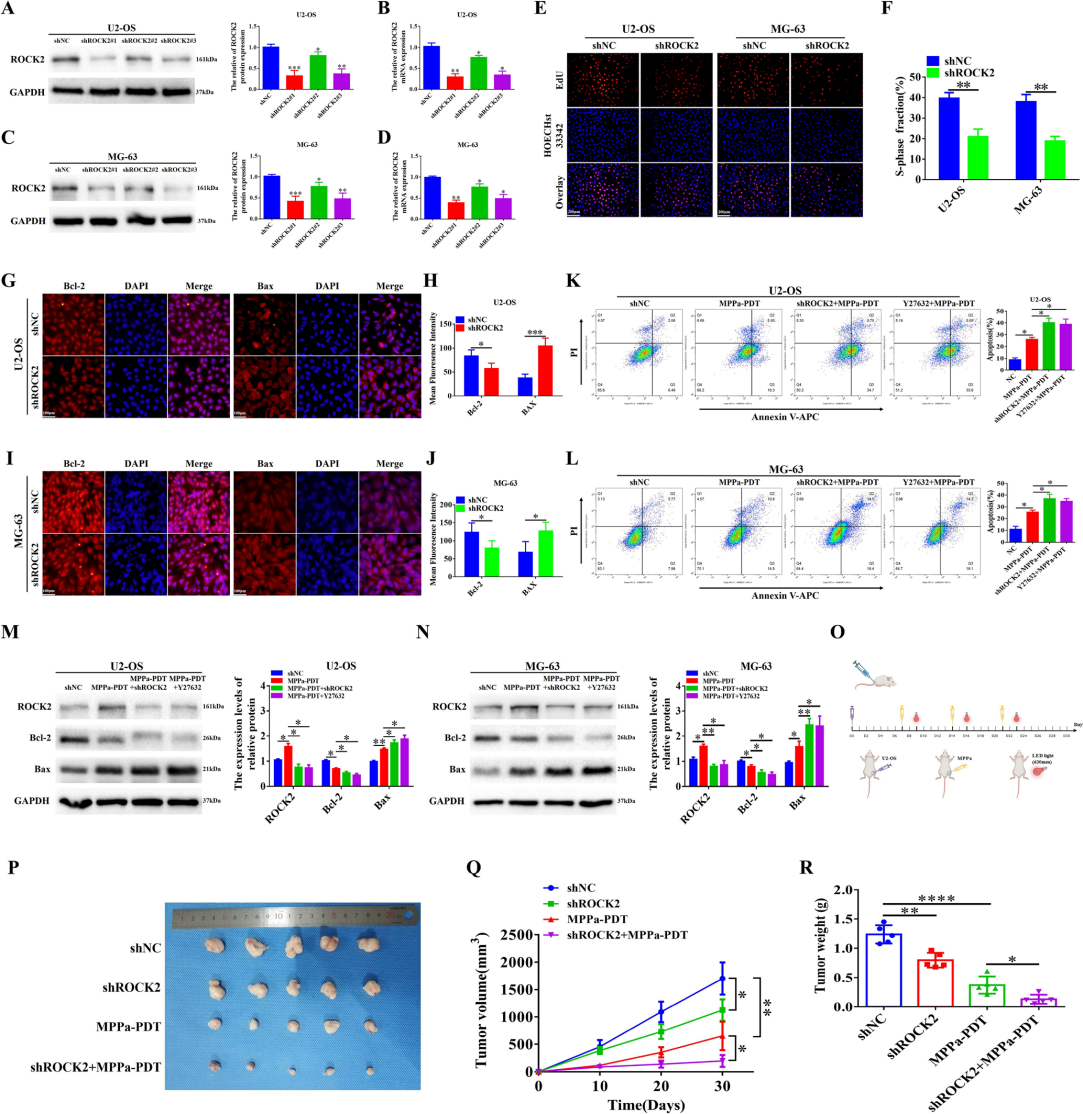
Inhibition of ROCK2 leads to sensitivity of OS to MPPa‐PDT in vivo and in vitro. (A–D) Western blot and qRT‐PCR were used to assess changes in protein and mRNA levels of ROCK2 in U2‐OS and MG‐63 cells after transfection with control shNC or shRNA targeting ROCK2. (E, F) EdU analysed the proliferation ability of U2‐OS and MG‐63 cells after transfection with shNC or shROCK2 (Scale bar: 100 μm). (G–J) Cell immunofluorescence analysis was used to detect the Bcl‐2 and BAX protein levels in U2‐OS and MG‐63 cells after transfection with shNC or shROCK2 (Scale bar: 200 μm). (K, L) MPPa‐PDT and shROCK2 or Y27632 were combined to treat U2‐OS and MG‐63 cells, and the apoptosis rate was detected by flow cytometry. (M, N) MPPa‐PDT combined with shROCK2 or Y27632 treated U2‐OS and MG‐63 cells, and Western blot detected the expression levels of apoptosis‐related proteins. (O) Process for constructing an OS xenograft model in nude mice. (P) U2‐OS cells with/without shROCK2 were injected into nude mice, and combined with/without MPPa‐PDT. Representative images of xenograft tumours in nude mice (*n* = 5). (Q, R) Tumour volume was measured every 10 days and corresponding tumour growth curves were obtained. Measurement of tumour weight in each nude mouse. *p < 0.05, **p < 0.01, ***p < 0.001, ****p < 0.0001.

PDT is a new method for the clinical treatment of OS following surgery, radiotherapy, and chemotherapy. However, PDT resistance in OS cells greatly limits the effects of PDT, and the specific underlying mechanism remains unclear. Therefore, we focused on the specific role of ROCK2 in the resistance of OS to MPPa‐PDT. We observed changes in the sensitivity of OS cells to MPPa‐PDT by changing the expression level of ROCK2. We first verified the efficiency of lentivirus that stably inhibited ROCK2 expression using Western blot and qRT‐PCR. The data showed that ROCK2 protein and mRNA levels were significantly reduced in OS cells, and the shROCK2#1‐lentivirus was selected for subsequent experiments (Figure 2A–D). We then used EdU to detect the changes in cell proliferation ability after inhibiting ROCK2 and found that this ability was weakened (Figure 2E,F). Furthermore, we verified the effect of altering ROCK2 expression on apoptosis using cell immunofluorescence. The results revealed that after the downregulation of ROCK2, the mean fluorescence intensity of Bcl‐2 protein in cells decreased, while the mean fluorescence intensity of Bax protein increased (Figure 2G–J). These results indicate that the apoptosis level of OS cells increased after downregulation of ROCK2 expression.

Next, we combined MPPa‐PDT with shROCK2‐lentivirus or Y27632, and the apoptosis rate of OS cells was determined using flow cytometry. Our data showed that the apoptosis rate of OS cells treated with MPPa‐PDT was significantly upregulated after ROCK2 inhibition (Figure 2K,L). Furthermore, we verified the changes in apoptosis‐related protein levels by Western blot. The results showed that the inhibition of ROCK2 expression significantly increased the levels of Bax protein, whereas the levels of Bcl‐2 protein were significantly downregulated after MPPa‐PDT treatment (Figure 2M,N). These results also indicate that inhibiting ROCK2 expression increases apoptosis in OS cells treated with MPPa‐PDT. The above results show that ROCK2 inhibition sensitises OS cells to MPPa‐PDT.

To further confirm that ROCK2 can lead to OS resistance to MPPa‐PDT, we verified whether inhibiting the expression of ROCK2 could reverse the tolerance of OS to MPPa‐PDT by subcutaneous xenogeneic tumour formation in BALB/c‐nude mice. We seeded OS cells subcutaneously into the right back of nude mice to construct a xenograft model to assess the effect of ROCK2 on MPPa‐PDT resistance in OS. We followed the process shown in Figure 2O to construct the xenograft model, including MPPa injection and LED light (630 nm) treatment. Our results showed that the tumour volume and weight in the shROCK2 and MPPa‐PDT groups were significantly smaller than those in the shNC group, indicating that both ROCK2 inhibition and MPPa‐PDT treatment inhibited OS growth (Figure 2P–R). Meanwhile, compared with the MPPa‐PDT group, combined MPPa‐PDT and ROCK2 inhibition further inhibited the growth of subcutaneous tumours in nude mice (Figure 2P–R). The results showed that inhibition of ROCK2 sensitises OS to MPPa‐PDT. Taken together, our data suggest that ROCK2 induces OS resistance to MPPa‐PDT both in vivo and in vitro.

### 3.3 ROCK2 Regulates MPPa‐PDT Resistance in OS Through Cellular Autophagy

**FIGURE 3:**
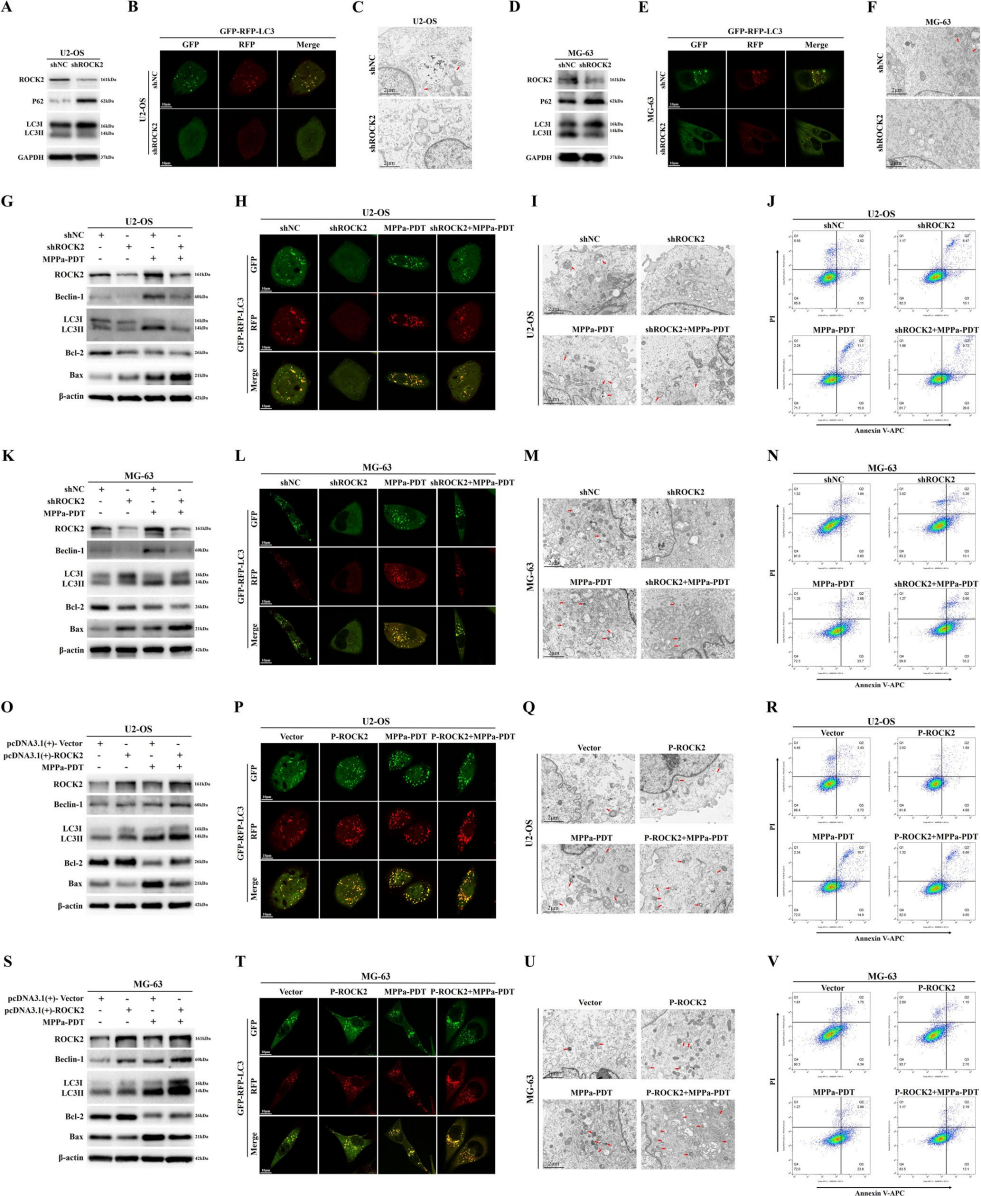
ROCK2 regulates MPPa‐PDT resistance in OS through cellular autophagy. (A, D) Western blot analysis of autophagy‐related protein levels in U2‐OS and MG‐63 cells after transfection with shROCK2. (B, E) Transfected mRFP‐GFP‐LC3 lentivirus labelled and tracked LC3. Autophagic flux and autophagy levels in U2‐OS and MG‐63 cells were monitored by confocal fluorescence microscope after transfection with shROCK2 (Scale bar: 10 μm). (C, F) Transmission electron microscope observed autophagosome‐like structures in U2‐OS and MG‐63 cells after transfection with shROCK2 (Scale bar: 2 μm). (G, K, O, S) Combined MPPa‐PDT and shROCK2 or pcDNA3.1(+)‐ROCK2 were used to treat OS cells, and the levels of ROCK2, autophagy‐associated proteins, and apoptosis‐associated proteins were detected by Western blot. (H, L, P, T) Cells were treated with a combination of MPPa‐PDT and shROCK2 or pcDNA3.1(+)‐ROCK2, and cellular autophagic flux and autophagy levels were monitored by confocal fluorescence microscope (Scale bar: 10 μm). (I, M, Q, M) Transmission electron microscope observed autophagosome‐like structures in OS cells after MPPa‐PDT combined with shROCK2 or pcDNA3.1(+)‐ROCK2 for treatment (Scale bar: 2 μm). (J, N, R, V) MPPa‐PDT was combined with shROCK2 or pcDNA3.1(+)‐ROCK2 to treat OS cells, and flow cytometry was used to detect the cell apoptosis rate.

This study demonstrated that ROCK2 can induce MPPa‐PDT resistance in OS; however, the mechanism by which ROCK2 mediates resistance remains unclear. Therefore, we further explored the specific mechanism by which ROCK2 leads to MPPa‐PDT resistance in OS. We first detected changes in the autophagy levels of OS cells following the downregulation of ROCK2 through Western blot. The results showed that the expression of P62 protein in OS cells increased after ROCK2 inhibition, whereas the ratio of LC3II/LC3I protein decreased, indicating that the level of autophagy in cells decreased (Figures 3A,D and S1A,D). In addition, we labelled and tracked LC3 by transfecting the mRFP‐GFP‐LC3 lentivirus and used confocal fluorescence microscopy to monitor changes in autophagic flux and autophagy levels in OS cells. We found that the levels of autophagosomes (yellow dots in the merged images) and autolysosomes (red dots in the merged images) in OS cells were reduced after inhibition of ROCK2 expression (Figures 3B,E and S1B,E). These data showed that autophagic flux and autophagy levels of OS cells were inhibited after inhibiting ROCK2. Further, we observed autophagosome‐like structures in OS cells by transmission electron microscope. Compared with the shNC group, the number of autophagosome‐like structures in the OS cells of the shROCK2 group was reduced, indicating that the level of autophagy was reduced (Figures 3C,F, S1F, and S3C). These results suggested that ROCK2 inhibition reduced autophagy in OS cells.

Next, we combined ROCK2 inhibition and MPPa‐PDT to treat OS cells and detected the levels of autophagy and apoptosis. First, Western blot showed that the LC3II/LC3I protein ratio and Beclin‐1 protein levels in cells decreased after ROCK2 inhibition, while both increased after MPPa‐PDT treatment. Compared with the control group, the expression of Bcl‐2 protein decreased in the ROCK2 inhibited and MPPa‐PDT groups, whereas the expression of Bax protein increased (Figures 3G,K and S1G,K). These results indicate that the autophagy level decreased after ROCK2 knockdown, whereas the level of autophagy increased after MPPa‐PDT treatment, and the apoptosis level increased in both groups. Furthermore, MPPa‐PDT was used in ROCK2 stably inhibited cells. The results showed that the ratio of LC3II/LC3I decreased compared to MPPa‐PDT treatment alone, and the expression level of Bcl‐2 protein further decreased, whereas the Bax protein level further increased (Figures 3G,K and S1G,K). This indicated that ROCK2 inhibition could downregulate autophagy levels in OS cells treated with MPPa‐PDT while further promoting apoptosis. Similarly, we monitored the autophagic flux and autophagy levels of OS cells that had been transfected with mRFP‐GFP‐LC3 lentivirus using confocal fluorescence microscopy. We found that the levels of autophagosomes and autolysosomes were reduced in OS cells treated with combined inhibition of ROCK2 and MPPa‐PDT compared with those treated with MPPa‐PDT alone (Figures 3H,L and S1H,L). These data also indicated that ROCK2 inhibition decreased autophagic flux and autophagy levels of OS cells treated with MPPa‐PDT. In addition, transmission electron microscope showed that the autophagosome‐like structures were decreased in the combined ROCK2 inhibition and MPPa‐PDT treatment groups compared to the MPPa‐PDT group, which also demonstrated that ROCK2 inhibition could decrease the autophagic activity of OS cells treated with MPPa‐PDT (Figures 3I,M and S1I,M). At the same time, we also verified through flow cytometry that ROCK2 inhibition based on MPPa‐PDT treatment increased the apoptotic level of OS (Figures 3J,N and S1J,N).

Next, we combined ROCK2 overexpression and MPPa‐PDT to treat OS cells. Western blot showed that autophagy increased, whereas apoptosis decreased after ROCK2 overexpression (Figures 3O,S and S1O,S). Then, in OS cells treated with MPPa‐PDT, ROCK2 overexpression further elevated the LC3II/LC3I protein ratio and Bcl‐2 levels, while reducing the Bax protein level (Figures 3O,S and S1O,S). These results indicate that overexpression of ROCK2 enhances autophagy in OS cells treated with MPPa‐PDT while reversing apoptosis induced by MPPa‐PDT. Meanwhile, confocal fluorescence microscopy revealed that, compared with OS cells in the MPPa‐PDT group, cells with further ROCK2 overexpression exhibited higher levels of autophagosomes and autolysosomes (Figures 3L,P and S1L,P). Transmission electron microscopy confirmed that the autophagosome‐like structures of OS cells in the P‐ROCK2 + MPPa‐PDT group were further increased compared with the MPPa‐PDT group (Figures 3Q,U and S1Q,U). These findings also indicate that ROCK2 overexpression further enhanced autophagy in OS cells following MPPa‐PDT treatment. In addition, flow cytometry revealed that ROCK2 overexpression reversed MPPa‐PDT‐induced apoptosis in OS cells (Figures 3R,V and S1R,V). In summary, we confirm that ROCK2 regulates MPPa‐PDT resistance in OS via cellular autophagy. MPPa‐PDT induces apoptosis in OS cells and the onset of cellular autophagy. ROCK2‐mediated autophagy reverses MPPa‐PDT‐induced apoptosis in OS cells. Overall, autophagy in OS cells in response to MPPa‐PDT treatment mainly plays a protective role.

### 3.4 Autophagy Plays a Critical Role in ROCK2‐Mediated Resistance to MPPa‐PDT in OS

**FIGURE 4:**
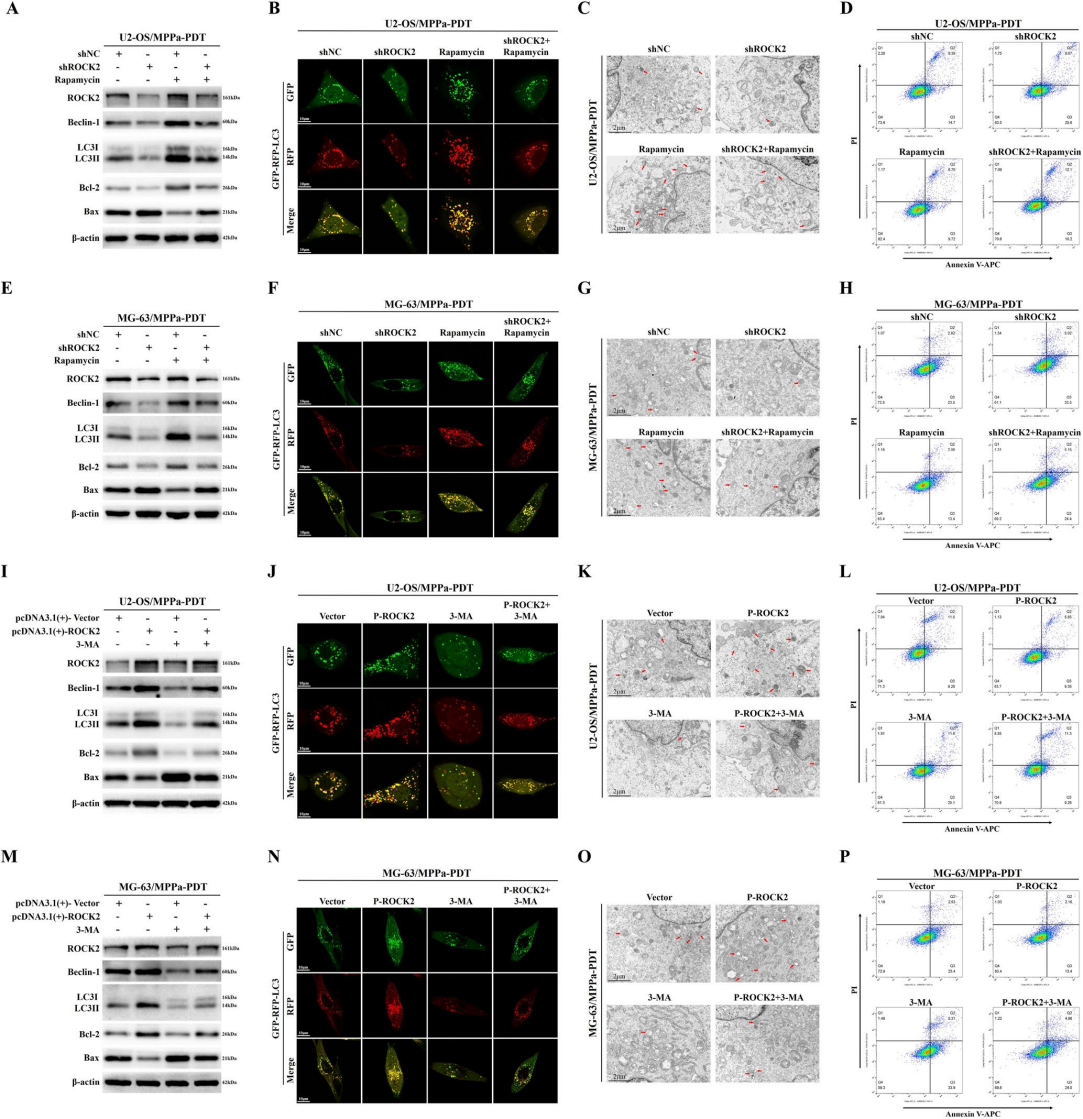
Autophagy plays a critical role in ROCK2‐mediated resistance to MPPa‐PDT in OS. (A, E) OS cells were treated with combined shROCK2 and Rapamycin under the condition of MPPa‐PDT treatment, and the levels of ROCK2, autophagy‐related proteins and apoptosis‐related proteins were detected by Western blot. (B, F) Combined treatment with shROCK2 and Rapamycin (100 nmol/L, 6 h) under MPPa‐PDT conditions and confocal fluorescence microscopy were used to monitor the autophagic flux and autophagy levels of OS cells (Scale bar: 10 μm). (C, G) Under the premise of MPPa‐PDT treatment, OS cells were treated with shROCK2 and Rapamycin, and intracellular autophagosome‐like structures were observed by transmission electron microscopy (Scale bar: 2 μm). (D, H) Based on MPPa‐PDT conditions, shROCK2 combined with Rapamycin acted on OS cells, and the apoptosis rate was detected by flow cytometry. (I, M) OS cells were treated with combined pcDNA3.1(+)‐ROCK2 and 3‐MA (5 mM, 2 h) under the condition of MPPa‐PDT treatment, and the levels of ROCK2, autophagy‐related proteins and apoptosis‐related proteins were detected by Western blot. (J, N) Combined treatment with pcDNA3.1(+)‐ROCK2 and 3‐MA under MPPa‐PDT conditions and confocal fluorescence microscopy were used to monitor the autophagic flux and autophagy levels of OS cells (Scale bar: 10 μm). (K, O) Under the premise of MPPa‐PDT treatment, OS cells were treated with pcDNA3.1(+)‐ROCK2 and 3‐MA, and intracellular autophagosome‐like structures were observed by transmission electron microscopy (Scale bar: 2 μm). (L, P) Based on MPPa‐PDT conditions, pcDNA3.1(+)‐ROCK2 combined with 3‐MA acted on OS cells, and the apoptosis rate was detected by flow cytometry.

To further investigate whether autophagy plays a key role in ROCK2‐mediated MPPa‐PDT resistance in OS, we first treated OS cells with a combination of ROCK2 inhibition along with the autophagy agonist‐Rapamycin on the premise of MPPa‐PDT treatment. Western blot revealed that Rapamycin treatment alone increased the levels of Beclin‐1, LC3II/LC3I ratio, and Bcl‐2, whereas those of Bax protein decreased. Compared to the ROCK2 downregulated group, further treatment with Rapamycin rescued the levels of Beclin‐1, LC3II/LC3I ratio, and Bcl‐2 which had been reduced by ROCK2 inhibition, while Bax protein level was also reduced instead (Figures 4A,E, S1E, and S2A). These data suggest that Rapamycin inhibits MPPa‐PDT‐induced apoptosis by enhancing cellular autophagy. Moreover, enhancing cellular autophagy reverses the further increase of apoptosis induced by ROCK2 downregulation in MPPa‐PDT‐treated cells. Similarly, confocal fluorescence microscope revealed elevated levels of intracellular autophagosomes and autolysosomes after Rapamycin treatment compared with those in the control group. Rapamycin treatment rescued the reduction in autophagosome and autolysosome levels induced by ROCK2 inhibition in MPPa‐PDT‐treated cells (Figures 4B,F and S2B,F). Additionally, transmission electron microscope revealed elevated intracellular levels of autophagosome‐like structures in the Rapamycin‐treated group compared to those in the control group. Rapamycin treatment also rescued the decline in autophagosome‐like structures due to ROCK2 inhibition in MPPa‐PDT‐treated cells (Figures 4C,G and S2C,G). In addition, flow cytometry similarly demonstrated that Rapamycin inhibited apoptosis in OS cells induced by MPPa‐PDT. Rapamycin also reversed the further increase in apoptosis in MPPa‐PDT‐treated cells caused by reduced ROCK2 expression (Figures 4D,H and S2D,H).

Next, OS cells were treated with the autophagy inhibitor‐3‐MA in combination with ROCK2 overexpression on the basis of MPPa‐PDT treatment. Western blot revealed that Beclin‐1, LC3II/LC3I ratio, and Bcl‐2 protein levels in OS cells reduced after treatment with 3‐MA compared to those in the MPPa‐PDT control group, while increasing Bax protein level (Figures 4I,M and S2I,M). 3‐MA effectively reversed the increase in Beclin‐1, LC3II/LC3I ratio, and Bcl‐2 protein levels in MPPa‐PDT‐treated OS cells due to ROCK2 overexpression, whereas the level of Bax protein was instead increased (Figure 4I,M and S2I,M). Furthermore, confocal fluorescence microscopy revealed that 3‐MA reduced the number of autophagosomes and autolysosomes in MPPa‐PDT‐treated OS cells. 3‐MA reversed the increased levels of autophagosomes and autolysosomes in MPPa‐PDT‐treated OS cells induced by ROCK2 overexpression (Figure 4J,N and S2J,N). Transmission electron microscopy also revealed a reduction in autophagosome‐like structures in MPPa‐PDT‐treated OS cells after further treatment with 3‐MA. 3‐MA reversed the increase in autophagosome‐like structures caused by ROCK2 overexpression in MPPa‐PDT‐treated OS cells (Figure 4K,O and S2K,O). Flow cytometry further demonstrated that 3‐MA promoted apoptosis in MPPa‐PDT‐treated OS cells and rescued the decrease in apoptosis induced by ROCK2 overexpression in MPPa‐PDT‐treated cells (Figure 4L,P and S2L,P). These results suggest that inhibiting cellular autophagy can further enhance apoptosis induced by MPPa‐PDT treatment. Inhibition of cellular autophagy rescued the reduction in apoptosis induced by ROCK2 overexpression in MPPa‐PDT‐treated OS cells. In conclusion, we can confirm that autophagy plays a critical role in ROCK2‐mediated resistance to MPPa‐PDT in OS and that it mediates MPPa‐PDT resistance by regulating cellular autophagy.

### 3.5 ROCK2 Regulates Autophagy Level in OS Cells via the Hippo Signalling Pathway

**FIGURE 5:**
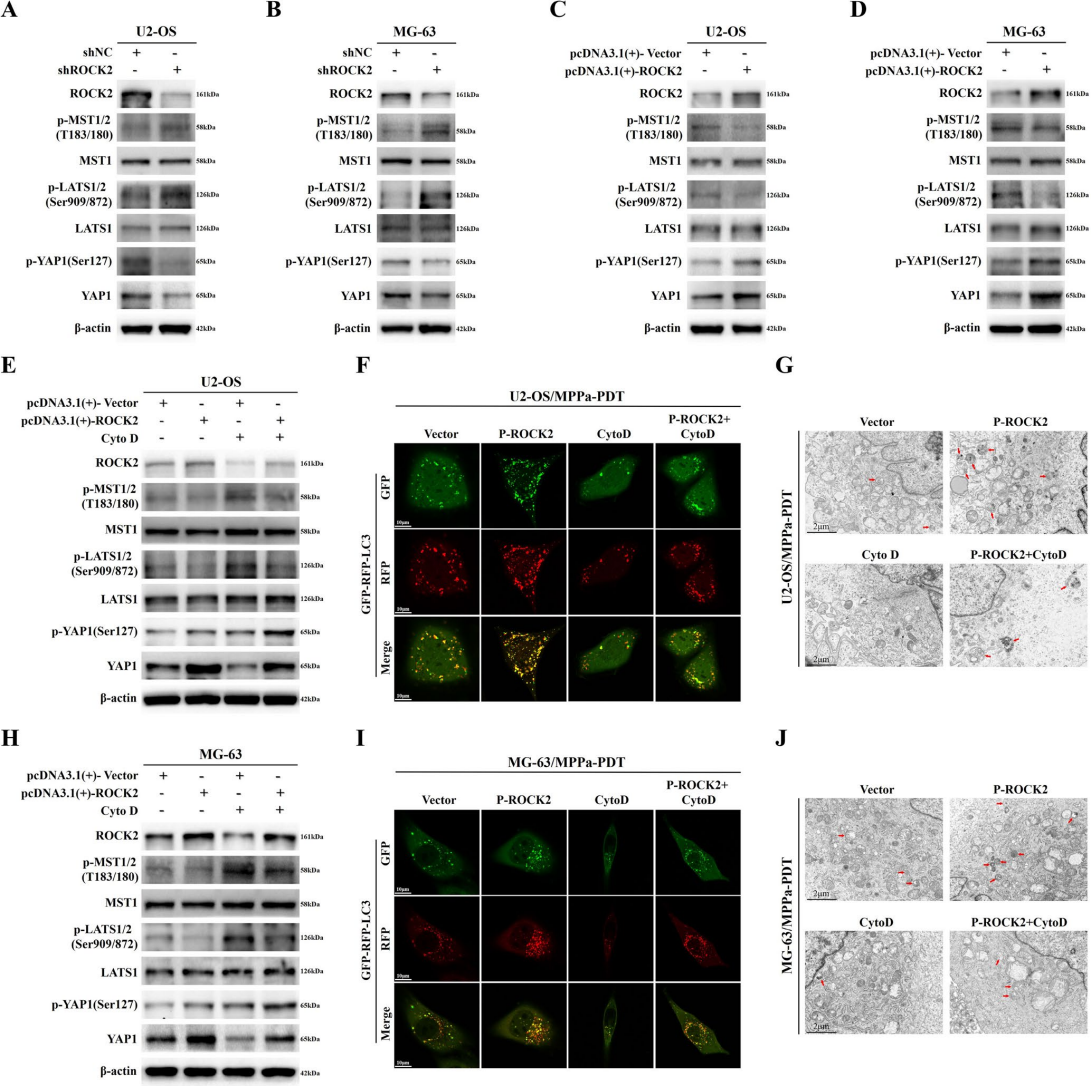
ROCK2 regulates autophagy level in OS cells via the Hippo signalling pathway. (A, B) Western blot detected the levels of ROCK2 and Hippo pathway‐related proteins in U2‐OS and MG‐63 cells after transfection with shROCK2. (C, D) Western blot detection of ROCK2 and Hippo pathway‐related protein levels in U2‐OS and MG‐63 cells after transfection with pcDNA3.1(+)‐ROCK2. (E, H) Combined pcDNA3.1(+)‐ROCK2 and Cyto D (200 nM) to treat OS cells, and the levels of ROCK2 and Hippo pathway‐related proteins were detected by Western blot. (F, I) OS cells were treated with combined pcDNA3.1(+)‐ROCK2 and Cyto D under MPPa‐PDT conditions, and autophagic flux and autophagy levels were monitored by confocal fluorescence microscope (Scale bar: 10 μm). (G, J) OS cells were treated with combined pcDNA3.1(+)‐ROCK2 and Cyto D under MPPa‐PDT treatment conditions, and autophagosome‐like structures were observed by transmission electron microscope (Scale bar: 2 μm).

We showed that ROCK2 can affect autophagy in OS cells, leading to resistance to MPPa‐PDT. However, the specific mechanism by which ROCK2 regulates autophagy remains unclear. Therefore, we first altered the ROCK2 levels to observe changes in the Hippo signalling pathway‐related proteins. After ROCK2 expression inhibition, Western blot revealed no significant changes in the total protein levels of MST1 and LATS1, whereas the protein levels of p‐MST1/2 (T183/180) and p‐LATS1/2 (Ser909/872) increased (Figures 5A,B and S3A,B). In addition, YAP1 and p‐YAP1 (Ser127) protein levels were reduced (Figures 5A,B and S3A,B). We subsequently overexpressed ROCK2, and Western blot showed no significant differences in the total protein levels of MST1 and LATS1 (Figures 5C,D and S3C,D). However, the protein levels of p‐MST1/2 (T183/180) and p‐LATS1/2 (Ser909/872) decreased, while those of YAP1 and p‐YAP1 (ser127) increased (Figures 5C,D and S3C,D). These results indicate that inhibition of ROCK2 activates the Hippo signalling pathway, whereas its overexpression inhibits the pathway. The decrease in p‐YAP1 (Ser127) protein levels after ROCK2 inhibition may be attributed to the activation of the Hippo signalling pathway, leading to more YAP1 being phosphorylated and retained in the cytoplasm and then degraded by the ubiquitination proteasome system, resulting in a decrease in total YAP1 protein levels, which in turn leads to a decrease in p‐YAP1 (ser127) protein levels. Conversely, this also explains why p‐YAP1 (ser127) protein levels were elevated when ROCK2 was overexpressed.

Next, we overexpressed ROCK2 and used cytochalasin D (Cyto D) to potentiate Hippo pathway activity, and observed changes in Hippo pathway‐related proteins by Western blot to determine pathway activity [38]. We found that p‐MST1/2 (T183/180), p‐LATS1/2 (Ser909/872) and p‐YAP1 (Ser127) protein expression increased in OS cells after Cyto D treatment, while YAP1 protein levels decreased (Figures 5E,H and S3E,H). In addition, compared with the ROCK2 overexpression group, further treatment with Cyto D increased p‐MST1/2 (T183/180), p‐LATS1/2 (Ser909/872), and p‐YAP1 (ser127) protein levels, whereas YAP1 protein levels decreased (Figures 5E,H and S3E,H). These data indicate that it can rescue reduced Hippo pathway activity caused by ROCK2 overexpression.

Subsequently, we treated OS cells with MPPa‐PDT and observed changes in cellular autophagy after ROCK2 overexpression combined with Cyto D treatment altered the Hippo pathway activity. We found that Cyto D decreased the levels of autophagosomes and autolysosomes in OS cells (Figures 5F,I and S3F,I). Cyto D reversed the increase in autophagosome and autolysosome levels in MPPa‐PDT‐treated cells induced by overexpression of ROCK2 (Figures 5F,I and S3F,I). Moreover, the autophagy‐like structures in the cells observed by transmission electron microscope showed the same trend (Figures 5G,J and S3G,J). Taken together, we conclude that the Hippo pathway activity can affect autophagy in OS cells. ROCK2 regulates autophagy in OS cells via the Hippo signalling pathway.

### 3.6 ROCK2 Competes With MST1 to Bind SAV1 and Thereby Regulates the Hippo Signalling Pathway

**FIGURE 6:**
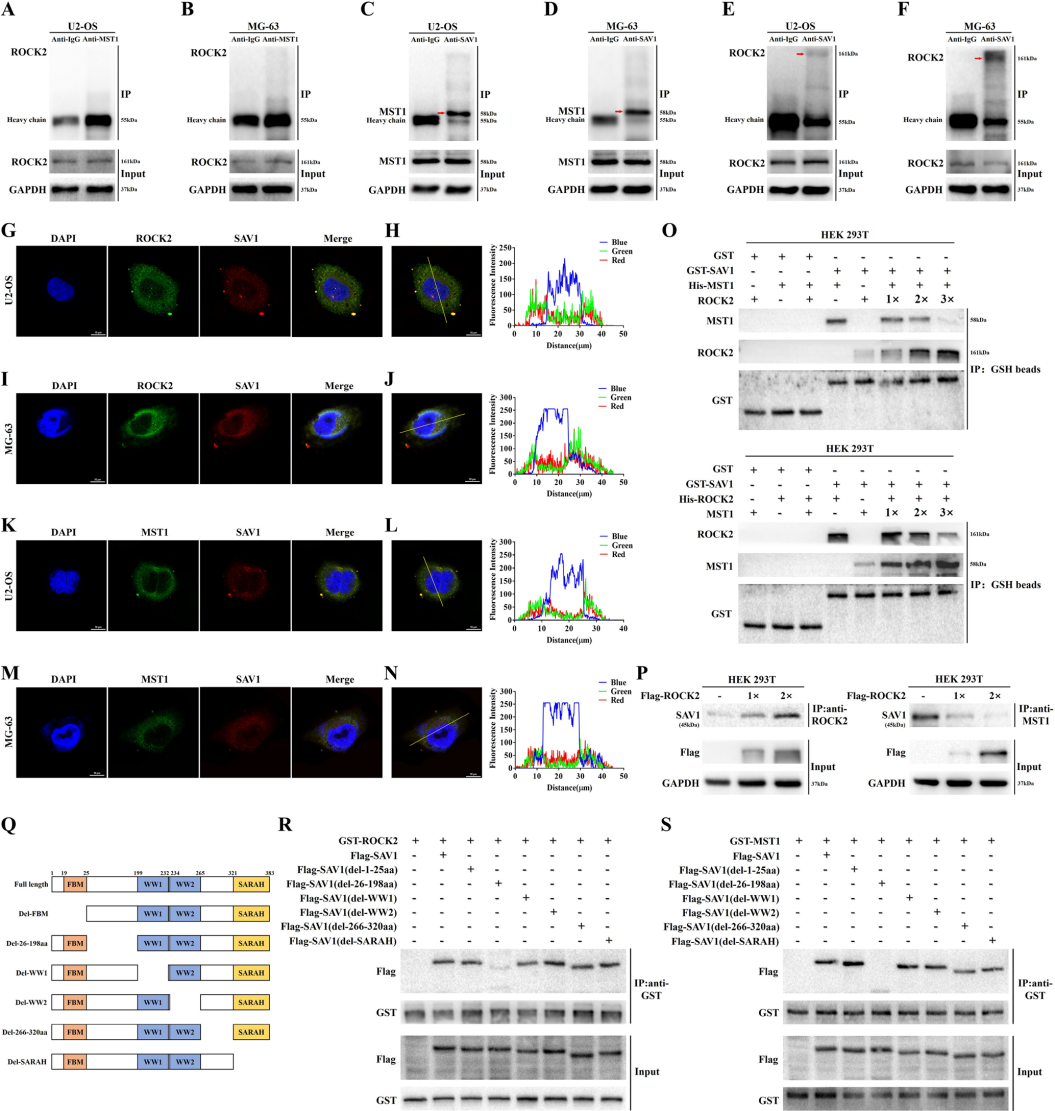
ROCK2 competes with MST1 to bind SAV1 and thereby regulates the Hippo signalling pathway. (A, B) Co‐IP analysis of endogenous MST1 and ROCK2 in OS cells. The IgG group was used as a control. (C, D) Analysis of endogenous SAV1 and MST in OS cells by Co‐IP. (E, F) Co‐IP analysis between endogenous SAV1 and ROCK2 in OS cells. (G, I) Immunofluorescence co‐localisation of ROCK2 and SAV1 in U2‐OS and MG‐63 cells (Scale bar: 10 μm). (H, J) Co‐localisation analysis of ROCK2 and SAV1 in U2‐OS and MG‐63 cells based on corresponding drawn lines. (K, M) Immunofluorescence co‐localisation of MST1 and SAV1 in U2‐OS and MG‐63 cells (Scale bar: 10 μm). (L, N) Co‐localisation analysis of MST1 and SAV1 in U2‐OS and MG‐63 cells based on corresponding drawn lines. (O) GST pulldown analysed SAV1 binding during the competition. (P) HEK 293T cells were transfected with increasing amounts of Flag‐ROCK2 plasmid. Cells were lysed and immunoprecipitated with anti‐ROCK2 and anti‐MST1 antibodies to detect endogenous SAV1 binding. (Q) Schematic representation of domains and motifs of human SAV1 and schematic representation of SAV1 mutations deleting different domains or fragments. (R, S) The constructed corresponding plasmid was transfected into HEK 293T. After cell lysis, anti‐GST beads were used to detect the Flag binding.

We have shown that ROCK2 can regulate the Hippo signalling pathway activity, but the underlying mechanism remains unclear. Therefore, we further investigated the specific mechanism by which ROCK2 regulates the Hippo pathway. We first analysed whether ROCK2 could bind to MST1, an upstream molecule in the core kinase chain of the Hippo pathway, using a Co‐IP assay and found that the two did not bind to each other (Figure 6A,B). SAV is a scaffold protein that can bind to MST1 and enhance its phosphorylation [39]. Therefore, we speculate that ROCK2 affects the levels of the MST1‐SAV1 complex by binding to SAV1, which in turn affects the phosphorylation of MST1, thereby regulating the Hippo pathway. We confirmed that ROCK2 binds to SAV1 protein using Co‐IP, while MST1 and SAV1 do bind to each other (Figure 6C–F). In addition, immunofluorescence co‐localisation and the statistical graph of co‐localisation analysis based on the corresponding drawn lines further corroborated that SAV1 proteins can bind to ROCK2 and MST1 proteins, respectively (Figure 6G–N). Next, to demonstrate the competitive binding between ROCK2 and MST1 for SAV1, a GST pulldown assay was conducted using HEK 293T cells, which confirmed their mutual competition for SAV1 binding (Figure 6O). Furthermore, our research showed that the levels of the ROCK2‐SAV1 complex gradually increased, whereas the levels of the MST1‐SAV1 complex gradually decreased with the progressive addition of ROCK2 plasmids in HEK 293T cells (Figure 6P). These experiments confirmed that ROCK2 competes with MST1 to bind SAV1 and that ROCK2 overexpression leads to an increase in ROCK2‐SAV1 complexes and a decrease in MST1‐SAV1 complexes.

Finally, we used HEK 293T cells to identify the sites where SAV1 binds to ROCK2 and MST1. Meanwhile, we further determined whether the two competed for binding to SAV1 at the same site. We divided SAV1 into different domains and fragments [40]. The different domains and fragments were then separately deleted and various corresponding plasmids were constructed (Figure 6Q). Co‐IP assays revealed that the aa 28–198 region of SAV1 contains the interaction region with ROCK2 and MST1, and that both compete for binding to SAV1 at the same sites (Figure 6R,S). In conclusion, these results indicate that ROCK2 binds competitively with MST1 to the aa 28‐198 region of SAV1, which in turn regulates the Hippo pathway.

### 3.7 Correlation Analysis of ROCK2 With Clinicopathological Features of OS and Its Association With the Expression of Molecules Related to the Hippo Pathway

**FIGURE 7:**
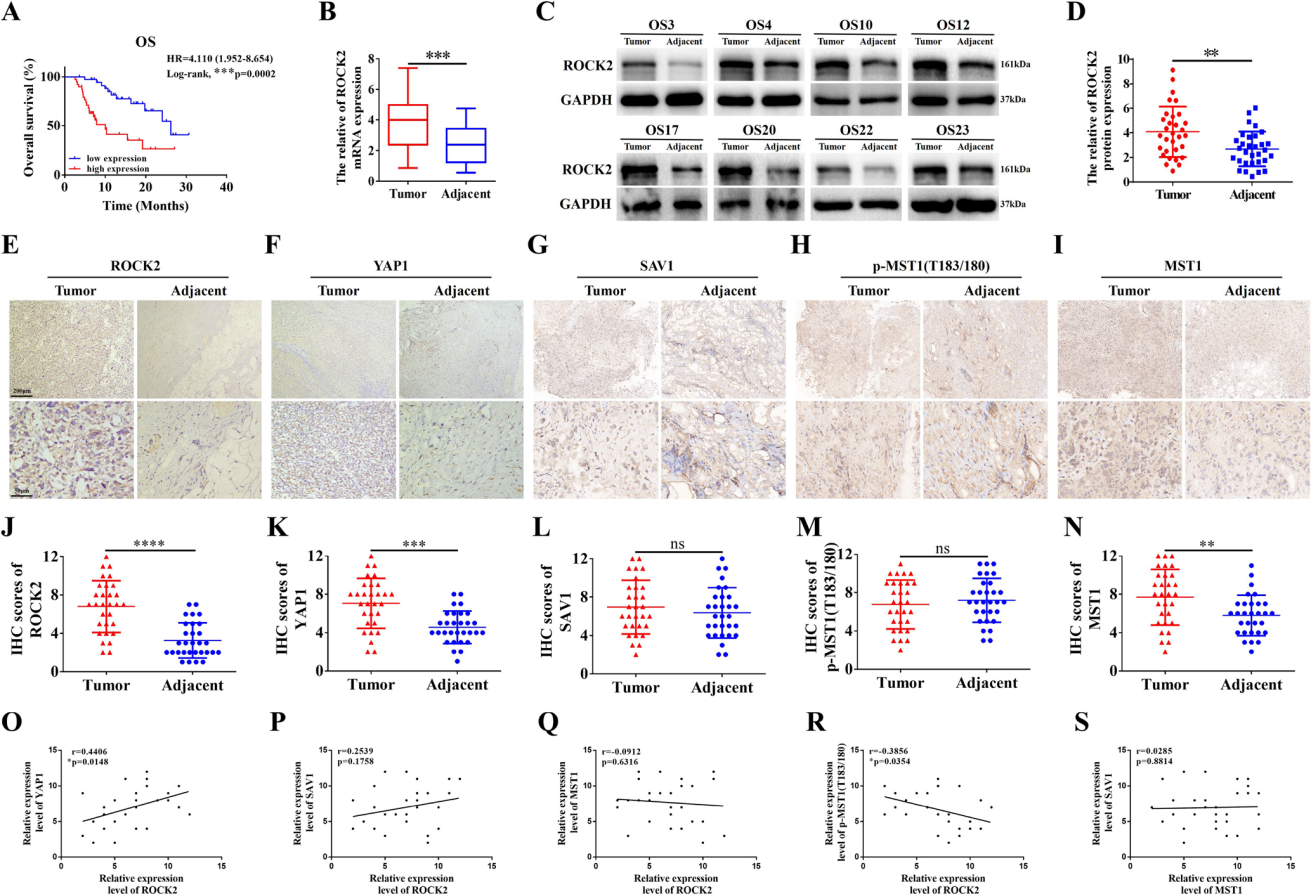
Correlation analysis of ROCK2 with clinicopathological features of OS and its association with the expression of molecules related to the Hippo pathway. (A) Kaplan–Meier analysis of the correlation between ROCK2 expression and overall survival in OS patients. (B–D) The mRNA and protein levels of ROCK2 in OS tissues (*n* = 30) and adjacent tissues (*n* = 30) were detected by qRT‐PCR and Western blot. (E–I) Representative images of IHC staining for ROCK2, YAP1, SAV1, p‐MST1(T183/180), and MST1 in OS tissues and their adjacent tissues (*n* = 30, Scale bar: 200 μm, 50 μm). (J–N) Statistical analysis of IHC staining scores for ROCK2, YAP1, SAV1, p‐MST1 (T183/180), and MST1. (O‐S) Correlation analysis based on IHC scores of associated proteins in OS tissues. *p < 0.05, **p < 0.01, ***p < 0.001, ****p < 0.0001.

We demonstrated that the regulation of ROCK2 on the Hippo pathway plays a pivotal role in OS resistance to MPPa‐PDT. Subsequently, we conducted a further analysis of ROCK2 based on the collected clinicopathological characteristics of patients with OS. We found that higher ROCK2 expression in OS was associated with larger tumour size, more metastases, and worse TNM stage (Table S1). Similarly, univariate and multivariate logistic regression analyses identified ROCK2 as an independent predictor of poor prognosis in patients with OS (Supplementary Table 2). In addition, our data also suggested that patients with high ROCK2 expression had worse overall survival than those with low ROCK2 expression by survival analysis using the Kaplan–Meier method (Figure 7A). Meanwhile, the validation of 30 randomly selected pairs of fresh OS tissues and their paired paracancerous tissues using qRT‐PCR and Western blot also reconfirmed that ROCK2 protein levels in OS tissues were significantly higher than those in paracancerous tissues (Figure 7B–D). These results showed that ROCK2 is closely related to the stage, progression, and prognosis of OS, supporting the significant role of ROCK2 in OS development. These results also highlight the potential of targeting ROCK2 as an effective therapeutic strategy for OS.

Next, we analysed the expression levels of ROCK2 and Hippo pathway‐related molecules in OS tissues by IHC. Our results showed that the expression levels of ROCK2, YAP1, and MST1 were higher in OS tissues than in paraneoplastic tissues, whereas the changes in the expression of SAV1 and p‐MST1 (T183/180) were not significant (Figure 7E–N). Furthermore, we performed a correlation analysis of the levels of ROCK2 and Hippo pathway‐related molecular proteins in OS tissues using IHC scores. Our data showed that ROCK2 expression in OS tissues positively correlated with YAP1 and negatively correlated with p‐MST1 (T183/180). Moreover, we found ROCK2 did not significantly correlate with SAV1 or MST1 expression. Concurrently, the expression of MST1 was not correlated with SAV1 (Figure 7O–S). The above results indicate a correlation between ROCK2 and the Hippo pathway and also corroborate that ROCK2 plays a pivotal role in OS‐associated phenotypes through the Hippo pathway from a clinical perspective, providing a potentially effective strategy and direction for the clinical treatment of OS.

### 3.8 J059‐0149 Reverses MPPa‐PDT Resistance in OS by Targeting ROCK2

**FIGURE 8:**
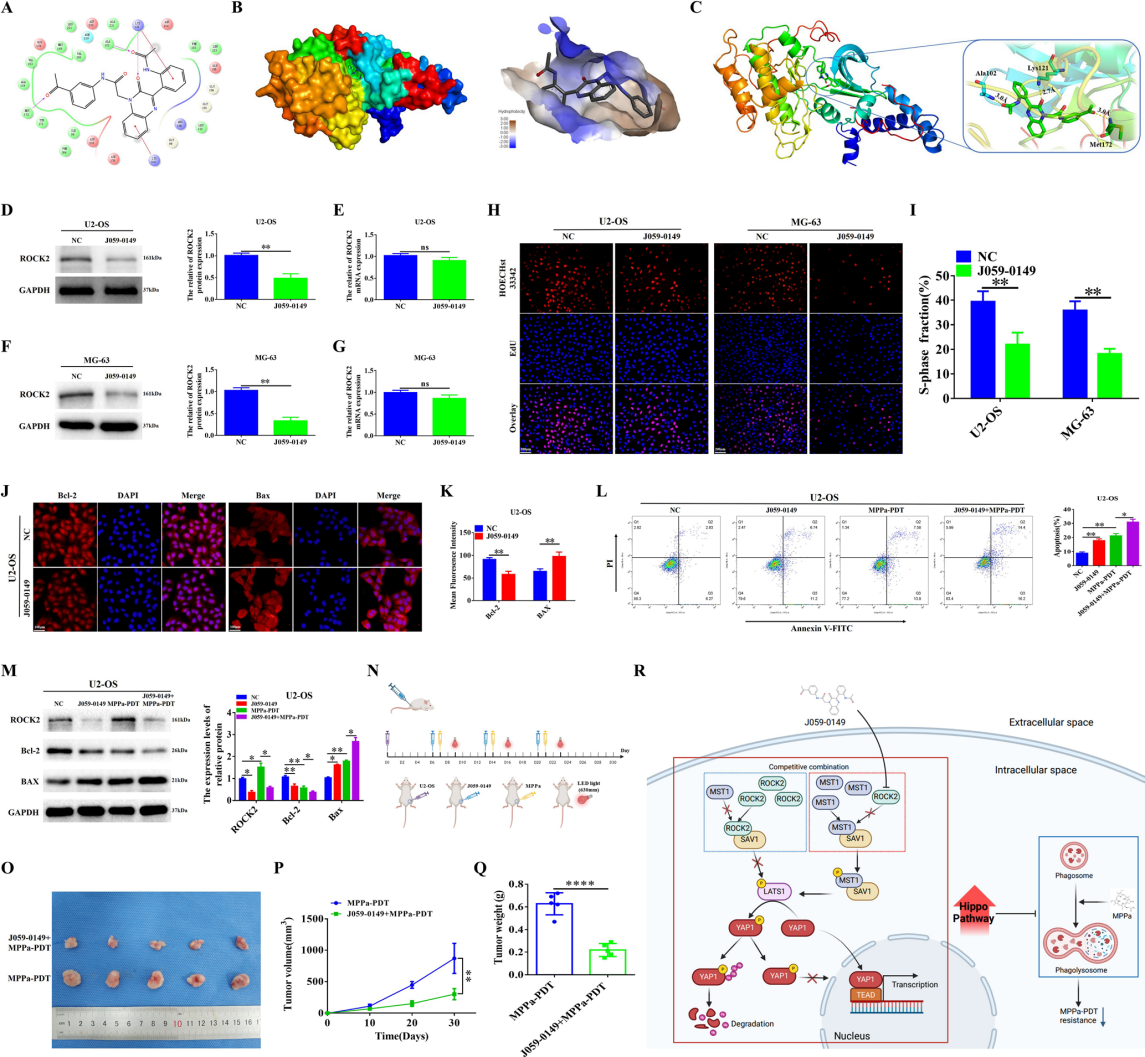
J059‐0149 reverses MPPa‐PDT resistance in OS by targeting ROCK2. (A) 2D diagram of the action force on compound J059‐0149. (B, C) Molecular docking predicts the binding mode and binding conformation of the ROCK2 protein structure to J059‐0149. (D–G) Western blot and qRT‐PCR assessed the protein and mRNA levels of ROCK2 in U2‐OS and MG‐63 cells treated with J059‐0149 (100 nM). (H, I) EdU was used to detect the proliferation ability of OS cells after J059‐0149 treatment (Scale bar: 100 μm). (J, K) Cell immunofluorescence analysis of Bcl‐2 and BAX protein levels in U2‐OS cells after J059‐0149 treatment (Scale bar: 200 μm). (L) Combined MPPa‐PDT and J059‐0149 were used to treat U2‐OS cells, and the apoptosis rate of the cells was detected by flow cytometry. (M) Combined MPPa‐PDT and J059‐0149 were used to treat U2‐OS cells, and the expression levels of apoptosis‐related proteins were detected by Western blot. (N) Processing procedures for constructing OS xenograft models in nude mice. (S) U2‐OS cells with/without J059‐0149 were injected into nude mice, and combined with/without MPPa‐PDT. Representative images of xenograft tumours in nude mice (*n* = 5). (O–Q) Tumour volume was measured every 10 days and corresponding tumour growth curves were obtained. Measurement of tumour weight in each nude mouse. (R) A model is proposed to reverse the resistance of osteosarcoma to MPPa‐PDT by targeting ROCK2 to activate the Hippo pathway and thereby inhibiting autophagy. *p < 0.05, **p < 0.01, ***p < 0.001, ****p < 0.0001. ns, nonsignificant.

Since ROCK2 plays a significant role in OS MPPa‐PDT resistance, targeting ROCK2 and reversing OS MPPa‐PDT resistance could be a potentially effective treatment. We performed virtual screening of 1.6 million compounds in the ChemDiv database based on molecular docking technology, followed by cascade molecular docking in high‐throughput virtual screening (HTVS), standard precision (SP), extra precision (XP) modes, combined with naked eye screening. Finally, the potential small‐molecule target compound J059‐0149, which had the highest score in the structural docking analysis with the ROCK2 protein, was screened for subsequent experimental analysis (Figure 8A–C). Western blot and qRT‐PCR confirmed that J059‐0149 effectively lowered the protein level of ROCK2 in OS cells, while mRNA expression showed no significant change (Figure 8D–G). EdU and cellular immunofluorescence analyses showed that treatment with J059‐0149 suppressed the proliferative ability of OS cells and increased their apoptosis (Figures 8H–K and S4A). Next, we combined MPPa‐PDT with J059‐0149 to treat the OS cells. Flow cytometry and Western blot analyses showed that J059‐0149 treatment resulted in increased levels of apoptosis in OS cells (Figures 8L,M and S4B,C). Compared to the MPPa‐PDT treatment group alone, the apoptosis level of OS cells treated with MPPa‐PDT increased even further after J059‐0149 inhibited ROCK2 protein level, indicating that these cells were more sensitive to MPPa‐PDT (Figures 8L,M and S4B,C). Furthermore, we assessed the effect on the MPPa‐PDT resistance of OS after ROCK2 inhibition using J059‐0149 by xenograft model in BALB/c‐nude mice. We followed the process shown in Figure 8R and processed accordingly and found that the volume and weight of tumours in the J059‐0149 + MPPa‐PDT group were significantly smaller than those in the MPPa‐PDT group, suggesting that the targeted inhibition of ROCK2 by J050‐0149 could lead to OS sensitivity to MPPa‐PDT (Figure 8O–Q). In conclusion, our data suggested that J059‐0149 can target and inhibit ROCK2 to reverse OS resistance to MPPa‐PDT. Targeting ROCK2 to reverse MPPa‐PDT resistance in OS is a potentially efficient therapeutic approach for clinical treatment.

## 4 Discussion

OS is a common primary bone tumour in children and adolescents, characterised by the formation of immature bone or bone‐like tissue from tumour cells with peritumour osteolysis [41]. OS commonly arises in rapidly growing bones, particularly in the epiphyses of long bones, such as the distal femur and proximal tibia [42]. The diagnosis of OS is based on clinical manifestations such as pain and swelling of the affected bone and is confirmed by radiography, CT, and tissue biopsy [43]. The survival rates for patients with OS remained relatively unchanged over the past decades, with a 5‐year survival rate of approximately 75% for patients with localised tumours, while it drops significantly to 20% for patients with a poor chemotherapy response or metastases [44, 45]. Therefore, there is an urgent clinical demand for novel and more effective therapies in addition to traditional surgery combined with neoadjuvant chemotherapy, to improve patient prognosis. PDT is a promising therapeutic strategy widely applied to various tumours, especially for the clinical treatment of superficial tumours due to its high targeting, minimally invasive, and low toxicity [46, 47]. The photosensitiser used in PDT therapy selectively accumulates at the tumour site and is excited by visible light irradiation at a specific wavelength to kill the tumour cells and tissues [48, 49]. This study aims to investigate the resistance of OS to MPPa‐PDT and further elucidate the underlying mechanisms.

Related studies have confirmed that MPPa‐PDT treatment induces increased levels of autophagy in OS cells, while studies have also demonstrated a close relationship between elevated cellular autophagy and chemoresistance to chemotherapeutic drugs [50, 51, 52]. Therefore, the presence of MPPa‐PDT resistance in OS may be attributed to an increased level of cellular autophagy during treatment, which may exert a protective effect on this process. Thus, the focus of this study was to investigate the role of autophagy in MPPa‐PDT resistance in OS and elucidate the specific mechanisms and key molecular targets that regulate autophagy. The aim is to provide an important theoretical basis for improving the therapeutic efficacy of MPPa‐PDT in clinical practice.

ROCK2 has been shown to be closely linked with autophagy and is capable of regulating the cellular level of autophagy [26, 53]. Our research team has dedicated several years to investigating the role of ROCK2 in tumour development, confirming its significance in these processes [54, 55]. Therefore, we explored whether ROCK2 regulates autophagy in OS cells and whether it is a pivotal target for reversing MPPa‐PDT tolerance in OS. We found that OS cell lines with higher ROCK2 expression were more resistant to MPPa‐PDT. Meanwhile, the RNA sequencing analysis showed that ROCK2 expression was increased. Moreover, Western blot and qRT‐PCR also observed an upregulation of ROCK2 expression in OS cells following MPPa‐PDT treatment. These results indicate that ROCK2 is closely related to the MPPa‐PDT resistance of OS. Next, our data showed that the apoptosis level of OS cells increased, and their proliferation ability was inhibited after ROCK2 inhibition. OS cells were more sensitive to MPPa‐PDT following the inhibition of ROCK2 expression. In vivo experiments further confirmed that ROCK2 inhibition led to enhanced OS sensitivity to MPPa‐PDT. These findings reinforce the crucial role of ROCK2 in promoting OS resistance to MPPa‐PDT. Subsequently, we further included autophagy in the study. Following downregulation of ROCK2 expression, reduced levels of cellular autophagy were observed. Therefore, we investigated whether ROCK2 affects MPPa‐PDT resistance in OS by regulating autophagy. We combined ROCK2 changes and MPPa‐PDT to treat OS cells and verified this speculation by detecting cellular autophagy and apoptosis levels. Our data indicate that ROCK2 mediates MPPa‐PDT resistance in OS by regulating cellular autophagy. Autophagy regulated by ROCK2 reverses the apoptosis of OS cells induced by MPPa‐PDT, providing a protective effect. To further clarify whether autophagy is the pivotal factor of ROCK2‐mediated MPPa‐PDT resistance in OS, we combined the alteration of ROCK2 expression and autophagy levels with Rapamycin or 3‐MA in the context of MPPa‐PDT treatment, as verified by rescue experiments. The data showed that the inhibition of autophagy rescued the decrease in apoptosis observed in MPPa‐PDT‐treated OS cells caused by ROCK2 overexpression. Conversely, increased autophagy reversed the elevated apoptosis induced by ROCK2 inhibition in MPPa‐PDT‐treated OS cells. Thus, we confirmed that autophagy is indeed a critical factor in ROCK2‐mediated MPPa‐PDT resistance in OS.

The Hippo pathway has been shown to be closely associated with the regulation of autophagy, and the degree of malignancy and chemoresistance of tumour cells can be regulated by functional crosstalk between the Hippo pathway and autophagy, suggesting that the Hippo pathway could be a suitable therapeutic target [32, 33, 56]. Therefore, we first sought to determine whether ROCK2 regulates the Hippo pathway. Our data suggest that ROCK2 inhibition results in the activation of the Hippo pathway, while conversely, overexpression of ROCK2 inhibits this pathway. Next, we used a combination of ROCK2 overexpression, cytochalasin D, and MPPa‐PDT to investigate whether the Hippo pathway is regulated by ROCK2 in OS cells and thus mediates autophagy. The corresponding experimental results corroborated this speculation. Subsequently, we explored the specific mechanisms by which ROCK2 regulates the Hippo pathway. Interestingly, experiments on protein interactions revealed that ROCK2 does not bind to MST1, an upstream molecule in the Hippo pathway, but binds to the scaffold protein SAV1. Studies have demonstrated that the binding of SAV1 to MST1 enhances the phosphorylation of MST1, thereby activating the Hippo pathway [39]. Consequently, ROCK2 might regulate the Hippo pathway by competing with MST1 for SAV1, thus affecting the phosphorylation level of MST1. We confirmed the correctness of this speculation through experiments such as GST pulldown. Subsequently, to investigate the specific sites of ROCK2 and MST1 competition for binding SAV1 and whether they are consistent, we divided SAV1 into different structural domains and truncated fragments, and constructed the corresponding plasmids with the corresponding deletions. The results of the corresponding experiments revealed that ROCK2 competes with MST1 to bind SAV1 at the same site, namely the aa 28‐198 region of SAV1.

This study mainly focuses on the effect of ROCK2 on MPPa‐PDT resistance in OS; therefore, we also analysed ROCK2 from a clinical perspective in patients with OS. Our data confirm that ROCK2 is associated with the clinicopathological features of patients with OS, is an independent predictor of poor prognosis, and that patients with high ROCK2 expression have worse overall survival than those with low ROCK2 expression. We also confirmed that ROCK2 expression was higher in OS tissues than in the adjacent tissues. These clinically relevant results strongly supported the conclusion that ROCK2 plays an important role in the development of OS. In addition, by analysing the expression levels of ROCK2 and Hippo pathway molecules in OS tissues, we confirmed their correlation. This further supports that ROCK2 plays a role in OS through the Hippo pathway and provides a potentially effective strategy and direction for the clinical treatment of OS. Therefore, we next started with the use of small molecule compounds targeting inhibition of ROCK2 to validate their effect on MPPa‐PDT resistance in OS. We screened the compounds in the ChemDiv database based on molecular docking technology and screened the targeted small‐molecule compound J059‐0149, which had the highest score in the structural docking analysis with the ROCK2 protein. We found that J059‐0149 significantly inhibited the protein expression of ROCK2 in OS cells, which was accompanied by the inhibition of cell proliferation and increased apoptosis. In vitro and *vivo* experiments showed that the inhibition of ROCK2 protein levels by J059‐0149 resulted in OS sensitivity to MPPa‐PDT. Thus, targeting ROCK2 for inhibition can reverse MPPa‐PDT resistance in OS and is a potentially effective clinical treatment.

In summary, ROCK2 competes with MST1 to bind to the aa 28–198 region of SAV1 and then regulates the Hippo pathway to affect autophagy, resulting in the resistance of OS to MPPa‐PDT. Moreover, we screened out J059‐0149, a small‐molecule compound targeting ROCK2 inhibition, and found that its inhibition of ROCK2 resulted in OS sensitisation to MPPa‐PDT (Figure 8R). In brief, our study confirmed that ROCK2 is a critical target for reversing MPPa‐PDT resistance and provides new targets and insights for the clinical treatment of MPPa‐PDT resistance in OS.

## Author Contributions

L.C. and L.H. conceived the research concept and design. L.H., L.C., and D.X. implemented the methodological development and drafted, reviewed, and revised the manuscript. X.Y., X.D., J.D., C.L., X.Y., X.H., S.C., and T.X. collected, analysed, and interpreted the data and conducted statistical analysis of the data; H.P., Y.D., Y.Z., and Q.L. extended material and technical support. All the authors read and authorised the final version of the paper.

## Ethics Statement

This study was approved by the Ethics and Research Committees of the Second Affiliated Hospital of Nanchang University. The animal work was approved by the Ethics Committee for Animal Experiments of the Second Affiliated Hospital of Nanchang University.

## Conflicts of Interest

The authors declare no conflicts of interest.

## Supporting information


**Supplementary Table S1.** Association of ROCK2 expression with clinical features in osteosarcoma patients.
**Supplementary Table S2.** Univariate and multivariate analysis of the prognostic factors in OS patients using a Cox regression model.
**Supplementary Figure S1.** (A, D) Statistical analysis of autophagy‐related protein levels detected by Western blot in U2‐OS and MG‐63 cells following transfection with shROCK2. (B, E, H, L, P, T) Perform statistical analysis on the total number of autophagosomes (yellow dots in the merged images) and autolysosomes (red dots in the merged images) in autophagy fluorescence images. (C, F, I, M, Q, U) Perform statistical analysis on the total numbers of autophagosomes (AP) and autolysosomes (AL) in transmission electron microscopy results from each experimental cell group. (G, K, O, S) Perform statistical analysis on the expression levels of ROCK2, autophagy‐associated proteins, and apoptosis‐associated proteins detected by Western blot. (J, N, R, V) Statistical analysis of apoptosis rate detected by flow cytometry. *p < 0.05, **p < 0.01, ***p < 0.001.
**Supplementary Figure S2.** (A, E, I, M) Perform statistical analysis on the expression levels of ROCK2, autophagy‐associated proteins, and apoptosis‐associated proteins detected by Western blot. (B, F, J, N) Perform statistical analysis on the total number of autophagosomes (yellow dots in the merged images) and autolysosomes (red dots in the merged images) in autophagy fluorescence images. (C, G, K, O) Perform statistical analysis on the total numbers of autophagosomes (AP) and autolysosomes (AL) in transmission electron microscopy results from each experimental cell group. (D, H, L, P) Statistical analysis of apoptosis rate detected by flow cytometry. *p < 0.05, **p < 0.01, ***p < 0.001. ns, nonsignificant.
**Supplementary Figure S3.** (A–D) Statistical analysis of Western blot detection for ROCK2 and Hippo signalling pathway‐associated protein levels in U2‐OS and MG‐63 cells. (E, H) OS cells were co‐treated with pcDNA3.1(+)‐ROCK2 and Cytochalasin D, statistical analysis was performed on protein expression levels of ROCK2 and Hippo pathway‐associated proteins detected by Western blot. (F, I) Perform statistical analysis on the total number of autophagosomes (yellow dots in the merged images) and autolysosomes (red dots in the merged images) in autophagy fluorescence images. (G, J) Statistical analysis of apoptosis rate detected by flow cytometry. *p < 0.05, **p < 0.01, ***p < 0.001. ns, nonsignificant.
**Supplementary Figure S4.** (A) Cell immunofluorescence analysis of Bcl‐2 and BAX protein levels in MG‐63 cells after J059‐0149 treatment (Scale bar: 200 μm). (B) Combined MPPa‐PDT and J059‐0149 were used to treat MG‐63 cells, and the apoptosis rate of the cells was detected by flow cytometry. **C**. Combined MPPa‐PDT and J059‐0149 were used to treat MG‐63 cells, and the expression levels of apoptosis‐related proteins were detected by Western blot. *p < 0.05, **p < 0.01.

## Data Availability

The data that support the findings of this study are available from the corresponding author upon reasonable request.
