# Supplementary material for: Reversal of MPPa‐PDT Resistance in Osteosarcoma by Targeting ROCK2‐Mediated Autophagy
Source: Cell Prolif. 2025 Jul 16;59(4):e70097. doi: 10.1111/cpr.70097 (PMC13052181; doi:10.1111/cpr.70097)
Supplement: Supplementary file 1 — Supplementary Table S1. Association of ROCK2 expression with clinical features in osteosarcoma patients. Supplementary Table S2. Univariate and multivariate analysis of the prognostic factors in OS patients using a Cox regression model. Supplementary Figure S1. (A, D) Statistical analysis of autophagy‐related protein levels detected by Western blot in U2‐OS and MG‐63 cells following transfection with shROCK2. (B, E, H, L, P, T) Perform statistical analysis on the total number of autophagosomes (yellow dots in the merged images) and autolysosomes (red dots in the merged images) in autophagy fluorescence images. (C, F, I, M, Q, U) Perform statistical analysis on the total numbers of autophagosomes (AP) and autolysosomes (AL) in transmission electron microscopy results from each experimental cell group. (G, K, O, S) Perform statistical analysis on the expression levels of ROCK2, autophagy‐associated proteins, and apoptosis‐associated proteins detected by Western blot. (J, N, R, V) Statistical analysis of apoptosis rate detected by flow cytometry. *p < 0.05, **p < 0.01, ***p < 0.001. Supplementary Figure S2. (A, E, I, M) Perform statistical analysis on the expression levels of ROCK2, autophagy‐associated proteins, and apoptosis‐associated proteins detected by Western blot. (B, F, J, N) Perform statistical analysis on the total number of autophagosomes (yellow dots in the merged images) and autolysosomes (red dots in the merged images) in autophagy fluorescence images. (C, G, K, O) Perform statistical analysis on the total numbers of autophagosomes (AP) and autolysosomes (AL) in transmission electron microscopy results from each experimental cell group. (D, H, L, P) Statistical analysis of apoptosis rate detected by flow cytometry. *p < 0.05, **p < 0.01, ***p < 0.001. ns, nonsignificant. Supplementary Figure S3. (A–D) Statistical analysis of Western blot detection for ROCK2 and Hippo signalling pathway‐associated protein levels in U2‐OS and MG‐63 cells. (E, H) OS cells w [file CPR-59-e70097-s001.docx]

**Supplementary Materials and Methods**

***Patients and clinical tissue samples***

OS tissues and their adjacent tissue specimens (78 cases) were collected from the Second Affiliated Hospital of Nanchang University, the First Affiliated Hospital of Nanchang University, and Jiangxi Cancer Hospital between October 2018 and October 2021. Informed consents were obtained from the patients and this study was approved by the Nanchang University Ethics Committee. None of the patients were receiving antitumour therapy at the same time of collection, and all specimens were confirmed by pathologists and stored in a ˗80 ℃ refrigerator for backup.

***Cell transfection***

ROCK2 overexpressed and down-regulated lentivirus, mRFP-GFP-LC3 lentivirus, and related plasmids were procured from GENECHEM (Shanghai, China). The amount of lentivirus required was calculated based on the measured Multiplicity of Infection (MOI). Transfection with a stable lentivirus was performed according to the manufacturer's instructions. Following 12–24 h of infection, fresh complete medium was added to maintain culture viability. Cell fluorescence was observed using a fluorescence microscope at 72 h post-transfection. Subsequently, the transfected cells were treated with puromycin (1–5 μg/ml, Sigma) for one week for cell clone screening after passaging. The screened cells were further validated and used for subsequent experiments.

***qRT-PCR***

Total RNA was extracted from tissues and cells using TRIzol reagent (Invitrogen, USA) according to the manufacturer's instructions. The purity and concentration of the extracted RNA were determined and used in subsequent experiments. Reverse transcription was performed using the Prime Script RT Reagent Kit (TaKaRa, Japan) according to the manufacturer’s instructions. Quantitative real-time polymerase chain reaction (qRT-PCR) was performed using a Real-Time PCR instrument (Applied Biosystems, USA) following the amplification procedure set up in the SYBR Premix Ex Taq II kit (TaKaRa, Japan) instructions. Finally, the values were statistically analyzed. The primer sequences are as follows: ROCK2 forward primer, 5'-GTCCGACCAGTTACACAGACA-3' and reverse primer 5'-CCAACTGGCTCCACTGGAAA-3'; GAPDH forward primer, 5'-GTTCGACAGTCAGCCGCATC-3' and reverse primer 5'-TGAAGGGGTCATTGATGGCA-3'.

***Western blot***

The collected tissues or cells were lysed by adding an appropriate amount of radioimmuneprecipitation assay buffer (RIPA, Applygen, China). The total protein concentration was measured using a BCA kit (Beyotime, China), and an appropriate amount of protein loading buffer (TransGen, China) was added before heating in boiling water for 10 min. Equal amounts of protein were separated by 10% sodium dodecyl sulfate-polyacrylamide gel electrophoresis (SDS-PAGE) and transferred onto polyvinylidene difluoride (PVDF, Millipore, USA) membranes. Skim milk was used to block membranes. Membranes incubated with the corresponding primary antibodies overnight at 4 ℃, followed by incubation with the secondary antibody (Boster, China)of the same species for 1 h. The membranes were washed with 1xTBST. Protein expression was visualized using Enhanced Chemiluminescence (ECL, Solarbio, China) and a chemiluminescent gel imaging analyzer (Bio-Rad, USA), and protein levels were quantified using Quantity One software (Bio-Rad, USA).

***Immunohistochemistry (IHC)***

The tissues were fixed, embedded in paraffin, sectioned, and subsequently incubated overnight at 4 °C with the corresponding primary antibodies at a concentration of 1:200. After washing with PBS, the secondary antibody was added and incubated for 2 h. Subsequently, labelling with DAB substrate from the EnVision HRP kit (DAKO, Denmark) was performed at room temperature for 30 min, followed by incubation and re-staining with Mayer's haematoxylin (DAKO, Denmark). Finally, sections were dehydrated, made transparent, sealed, and observed under a microscope.

***EdU***

The cells were seeded at a density of 1×10^4^ cells per well in 96-well plates, followed by the addition of 200 µL complete medium and incubation for 24 h prior to solution change. A total of 100 µL of 5-Ethynyl-2'-deoxyuridine (EdU) medium was added to each well following the kit (Ribobio, China) instructions, and incubated at 37℃ for 2 h. After removal of the medium, fixation with 4% paraformaldehyde (100 μL) was performed for 30 min followed by addition of glycine (2 mg/mL, 100 μL) and incubation at room temperature for 5 min. Subsequently, 100 μL of Apollo staining reaction solution was added and incubated for 30 min on a decolorization shaker under light-protected conditions. Next, 100 μL of 0.5% Triton-X was incubated for 20 min, followed by two washes with PBS and a half-hour incubated on a decolorization shaker with 100 μL of Hoechast33342 (5 mg/mL) protected from light. The samples were then washed twice with PBS and then observed under fluorescence microscopy.

***Flow cytometry***

Cells were harvested in flow tubes using 0.25% white trypsin, followed by low-speed centrifugation to remove the medium, and washed twice with pre-cooled PBS. The rate of apoptosis was evaluated via flow cytometry utilizing the apoptosis detection kit (KeyGEN, China).

***Analysis of autophagic flux***

The mRFP-GFP-LC3 lentivirus was transfected to label and track changes in cellular LC3 levels, enabling the monitoring of cellular autophagic flux. OS cells were transfected with mRFP-GFP-LC3 lentivirus, followed by corresponding downregulation of ROCK2 and MPPa-PDT treatment as required by the experiment. Cell fluorescence was observed using a confocal fluorescence microscope, with autophagosomes and autolysosomes represented by yellow and by red dots, respectively. Autophagic flux was assessed by analysing changes in fluorescence colour.

***Transmission electron microscope***

Cells were collected and fixed in electron microscope fixative (Servicebio, China) at 4℃ for 4 h, followed by rinsing with phosphate buffer (PB, pH 7.4). Subsequently, the cells were fixed with 1% osmium acid-PB at room temperature for 2 h and then rinsed three times with PB for 15 min each. The cells were then dehydrated using acetone and permeabilised overnight before embedding and sectioning (60–80 nm). The sections were then subjected to double staining with uranium-lead (2% uranyl acetate-saturated alcohol solution and lead citrate, each for 15 min), followed by overnight drying at room temperature. The sections were observed under a transmission electron microscope and images were collected for analysis.

***Xenograft model***

The 5-week-old female BALB/C nude mice used in the experiments were procured from Hunan SJA Laboratory Animal Co., Ltd. Prior to inoculation, the mice were acclimatised to under specific pathogen free (SPF) conditions for a week. The cells of each group treated accordingly were digested and resuspended, injected into the root of the right leg of nude mice at 1 × 10^6^ cells/100 μL PBS. Subsequent treatment was administrated as planned. The tumour size was measured periodically (calculated volume = shortest diameter/2 × longest diameter/2). Tumours were harvested from the anesthetized mice after 30 days, weighed individually, and photographed.

**Supplementary Table**

**Supplementary Table 1** Association of ROCK2 expression with clinical features in osteosarcoma patients.

| Classification | Total | ROCK2 expression | | χ^2^ | p-Value^a^ |
| --- | --- | --- | --- | --- | --- |
|  |  | high | low |  |  |
| **Age** |  |  |  | 0.053 | p=0.819 |
| < 18 | 45 | 22 | 23 |  |  |
| ≥ 18 | 33 | 17 | 16 |  |  |
| **Gender** |  |  |  | 1.857 | p=0.173 |
| Male | 36 | 21 | 15 |  |  |
| Female | 42 | 18 | 24 |  |  |
| **Tumor size (cm)**  < 5 28  ≥ 5 50  **Location**  Upper limb bone 35  Lower limb bone 43  **TNM stage** | | 8  31    20  19 | 20  19    15  24 | 8.023      1.296      13.570 | **p=0.005**      p=0.255      **p**<**0.001** |
| I/II | 32 | 8 | 24 |  |  |
| III/IV | 46 | 31 | 15 |  |  |
| **Distant metastasis** | |  |  | 7.389 | **p=0.007** |
| M0 | 40 | 14 | 26 |  |  |
| M1 | 38 | 25 | 13 |  |  |
| **Vital status** |  |  |  | 0.867 | p=0.352 |
| Alive | 48 | 22 | 26 |  |  |
| Dead | 30 | 17 | 13 |  |  |
| **Recurrence** |  |  |  | 1.877 | P=0.171 |
| Absence | 44 | 19 | 25 |  |  |
| Presence | 34 | 20 | 14 |  |  |

^a^ Chi-square test

**Supplementary Table 2** Univariate and multivariate analysis of the prognostic factors in OS patients using a Cox regression model.

| Parameters | Univariate analysis | | | | Multivariate analysis | | |
| --- | --- | --- | --- | --- | --- | --- | --- |
|  | HR | 95%CI | *p* value | HR | | 95%CI | *p* value |
| Age (<18 VS ≥ 18) | 1.22 | 0.61-2.44 | 0.583 | ­ ― | | ― | ― |
| Gender (Female VS male) | 0.86 | 0.43-1.73 | 0.674 | ― | | ― | ― |
| Tumor size (<5 VS ≥ 5)  Location (Upper limb VS Lower limb)  Stage (I/II VS III/IV) | 4.05  1.04  8.43 | 1.56-10.57  0.51-2.11  2.92-24.30 | **0.004****  0.920  **7.95E-05***** | 2.10  ―  2.91 | | 0.78-5.60  ―  0.85-9.94 | 0.140  ―  0.089 |
| Distant metastasis (M0 VS M1) | 7.47 | 2.87-19.47 | **3.82E-05***** | 4.53 | | 1.34-15.30 | **0.015*** |
| Recurrence (Absence VS presence) | 1.51 | 0.75-3.05 | 0.250 | 1.50 | | 0.62-3.62 | 0.364 |
| ROCK2 expression (High VS Low) | 1.13 | 1.07-1.20 | **4.31E-05***** | 1.09 | | 1.01-1.18 | **0.036*** |

HR, hazard ratio; CI, confidence interval; *****p<0.05; ******p<0.01; *******p<0.001.

**Supplementary Figure**


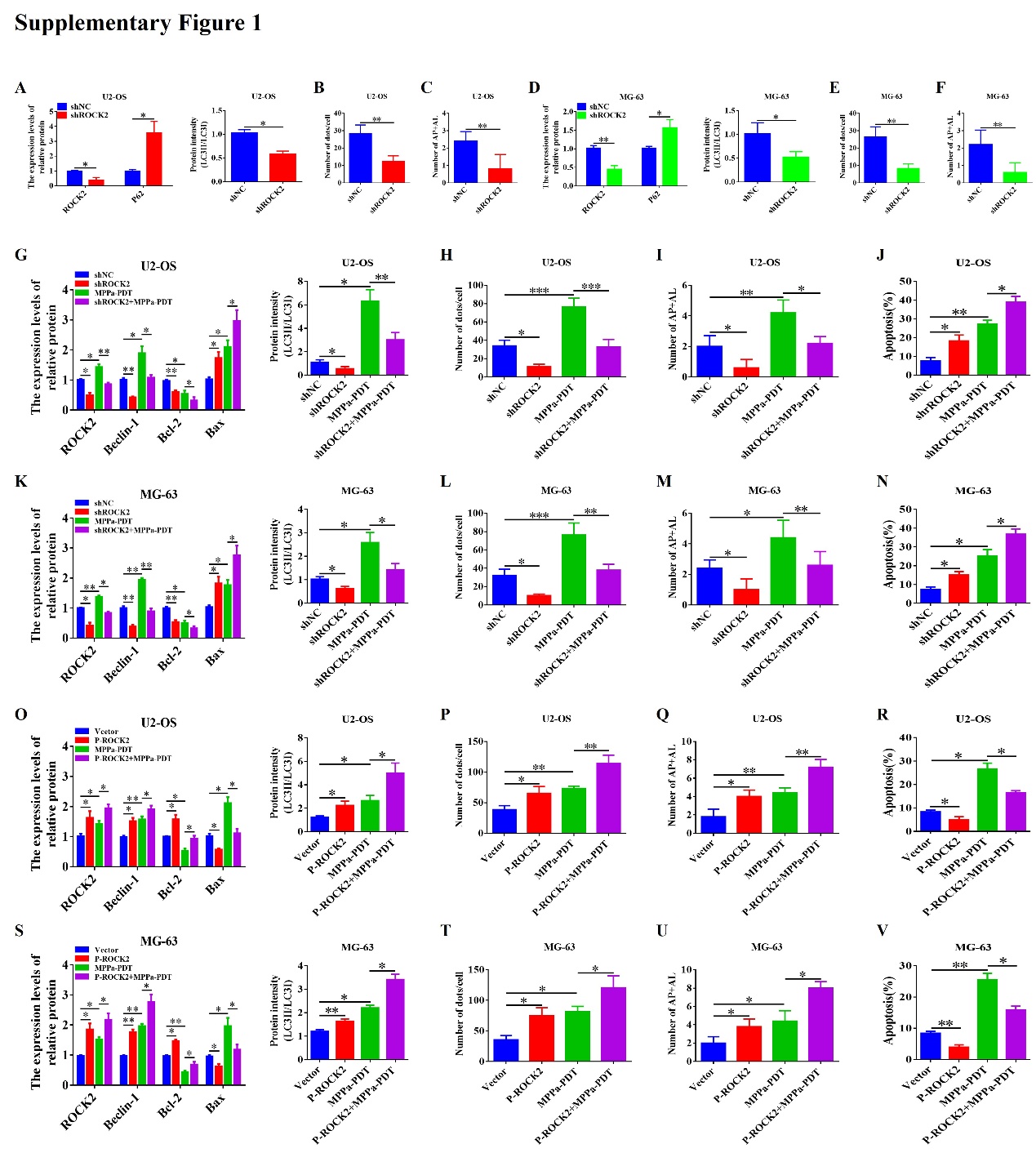


**Supplementary Figure 1.** **A, D.** Statistical analysis of autophagy-related protein levels detected by Western blot in U2-OS and MG-63 cells following transfection with shROCK2. **B, E, H, L, P, T.** Perform statistical analysis on the total number of autophagosomes (yellow dots in the merged images) and autolysosomes (red dots in the merged images) in autophagy fluorescence images. **C, F, I, M, Q, U.** Perform statistical analysis on the total numbers of autophagosomes (AP) and autolysosomes (AL) in transmission electron microscopy results from each experimental cell group. **G, K, O, S.** Perform statistical analysis on the expression levels of ROCK2, autophagy-associated proteins, and apoptosis-associated proteins detected by Western blot. **J, N, R, V.** Statistical analysis of apoptosis rate detected by flow cytometry. *p＜0.05, **p＜0.01, ***p＜0.001.


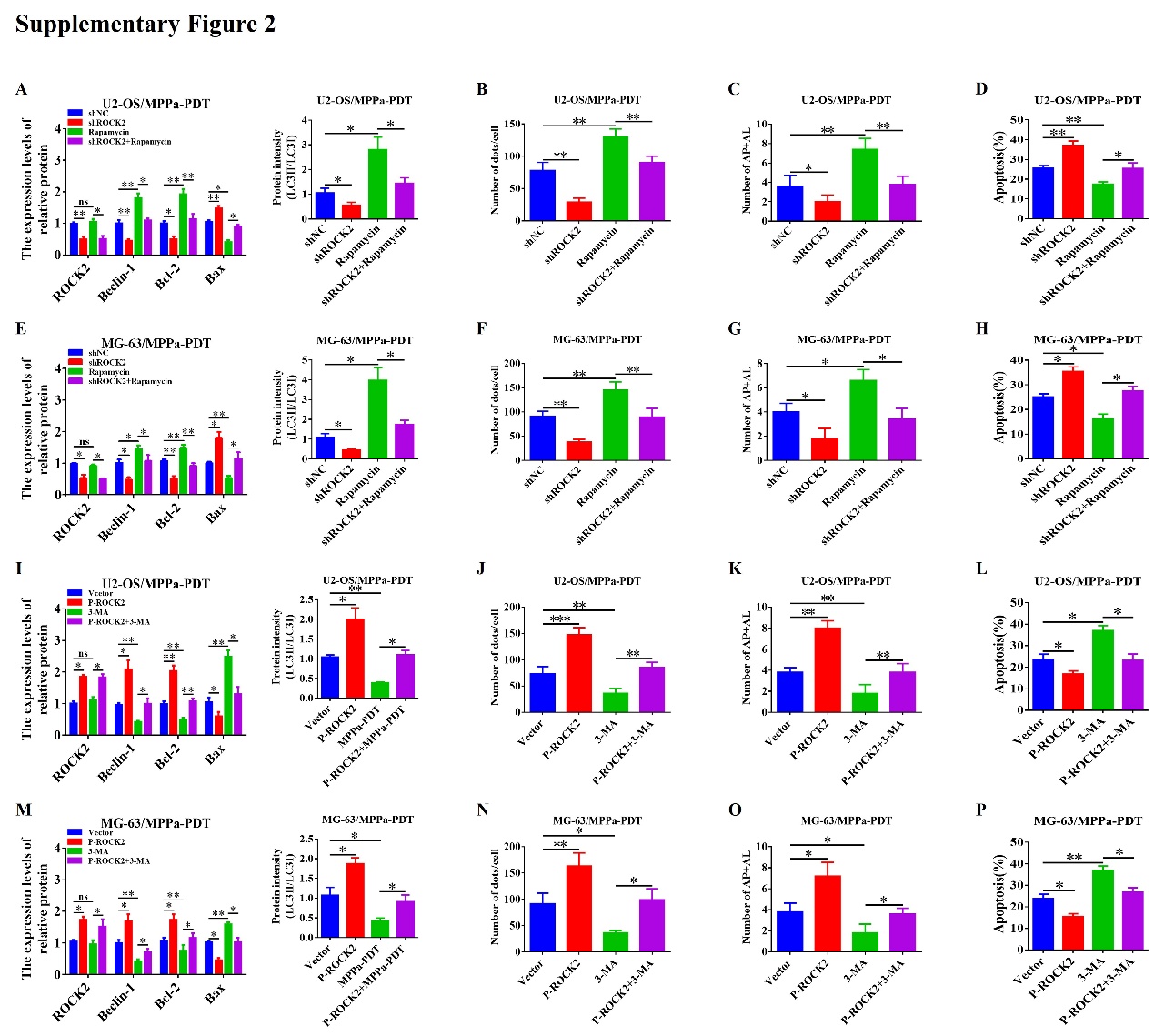


**Supplementary Figure 2. A, E, I, M.** Perform statistical analysis on the expression levels of ROCK2, autophagy-associated proteins, and apoptosis-associated proteins detected by Western blot. **B, F, J, N.** Perform statistical analysis on the total number of autophagosomes (yellow dots in the merged images) and autolysosomes (red dots in the merged images) in autophagy fluorescence images. **C, G, K, O.** Perform statistical analysis on the total numbers of autophagosomes (AP) and autolysosomes (AL) in transmission electron microscopy results from each experimental cell group. **D, H, L, P.** Statistical analysis of apoptosis rate detected by flow cytometry. *p＜0.05, **p＜0.01, ***p＜0.001. ns, nonsignificant.


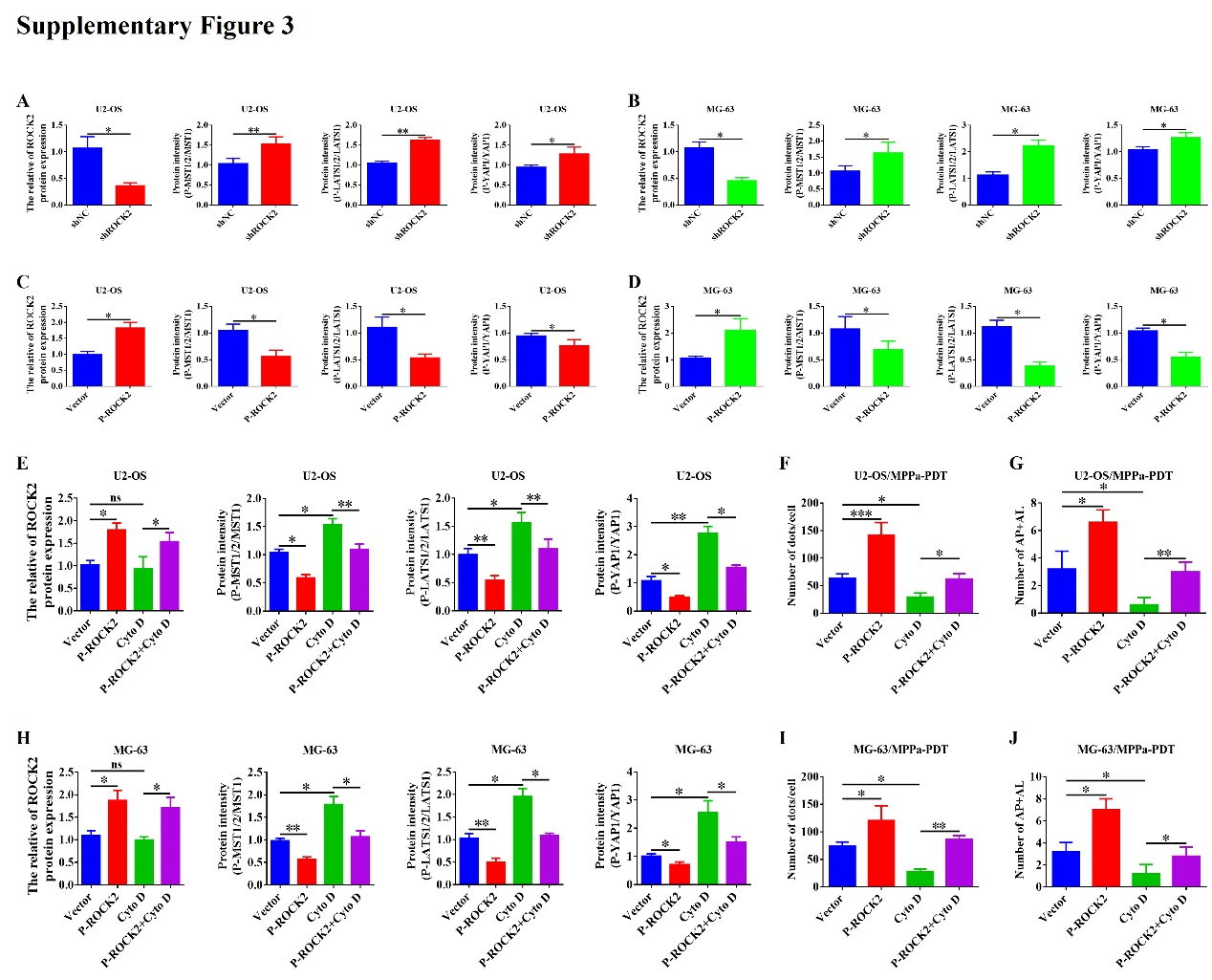


**Supplementary Figure 3. A-D.** Statistical analysis of Western blot detection for ROCK2 and Hippo signaling pathway-associated protein levels in U2-OS and MG-63 cells. **E, H.** OS cells were co-treated with pcDNA3.1(+)-ROCK2 and Cytochalasin D, statistical analysis was performed on protein expression levels of ROCK2 and Hippo pathway-associated proteins detected by Western blot. **F, I.** Perform statistical analysis on the total number of autophagosomes (yellow dots in the merged images) and autolysosomes (red dots in the merged images) in autophagy fluorescence images. **G, J.** Statistical analysis of apoptosis rate detected by flow cytometry. *p＜0.05, **p＜0.01, ***p＜0.001. ns, nonsignificant.


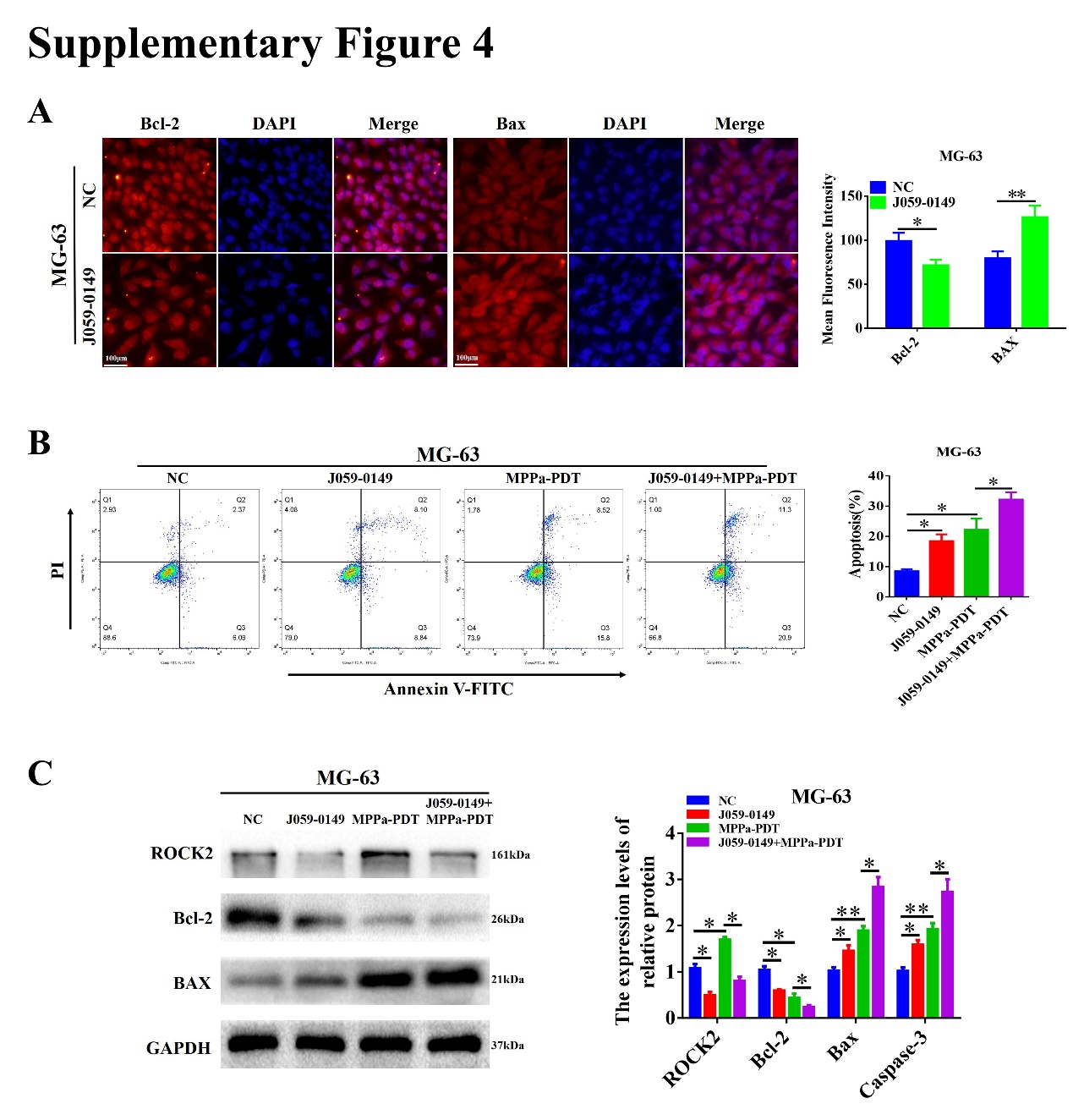


**Supplementary Figure 4.** **A.** Cell immunofluorescence analysis of Bcl-2 and BAX protein levels in MG-63 cells after J059-0149 treatment (Scale bar:200μm). **B.** Combined MPPa-PDT and J059-0149 were used to treat MG-63 cells, and the apoptosis rate of the cells was detected by flow cytometry. **C.** Combined MPPa-PDT and J059-0149 were used to treat MG-63 cells, and the expression levels of apoptosis-related proteins were detected by Western blot. *p＜0.05, **p＜0.01.
